# Human Microbiota-Associated Pig Models for Translational Microbiome Research: A Scoping Review

**DOI:** 10.3390/ijms27041987

**Published:** 2026-02-19

**Authors:** Seong-Jin Wang, Hao-Yang Nian, Zhi-Hao Chen, Li Cui

**Affiliations:** 1 Shanghai Key Laboratory of Veterinary Biotechnology, Shanghai Jiao Tong University, Shanghai 200240, China; jomin127@sjtu.edu.cn (S.-J.W.); nhy357@sjtu.edu.cn (H.-Y.N.); czhaoooo@sjtu.edu.cn (Z.-H.C.); 2 Department of Animal Science, School of Agriculture and Biology, Shanghai Jiao Tong University, Shanghai 200240, China; 3 Korea Disease Control and Prevention Agency (KDCA), Cheong-Ju 28159, Republic of Korea

**Keywords:** microbiome, microbiota, human microbiota-associated pig model, HMA pig model, fecal microbiota transplant, FMT, humanized pig model

## Abstract

The human microbiota-associated (HMA) pig model provides a physiologically relevant platform that bridges preclinical and translational research. However, its use remains limited, with existing studies showing considerable variation in establishment methods. This scoping review systematically evaluates methodological frameworks, engraftment outcomes, and research applications of HMA pig models. Additionally, it highlights their strengths, limitations, and implications for future studies. We conducted a comprehensive literature search in PubMed, Web of Science, Scopus, and Directory of Open Access Journals, following PRISMA guidelines for Scoping Reviews. The review examines the methodological foundations of HMA pig model generation and proposes a minimal reporting framework to promote standardization. It synthesizes studies on human microbiota engraftment in pigs, identifying factors that influence colonization efficiency. Finally, it summarizes current applications, discusses persistent limitations and translational challenges, and outlines opportunities for future research. Overall, these integrated insights aim to foster standardized, reproducible protocols for HMA pig model preparation and guide advancements in the field.

## 1. Introduction

The human microbiome comprises diverse microbial communities, including bacteria, archaea, eukaryotes, and viruses, that colonize body sites such as the skin, oral cavity, gut, respiratory tract, and urogenital system, along with their complete genetic material [1,2]. Joshua Lederberg introduced the human microbiome in 2001, sparking extensive research into its roles in human health and disease [3,4]. Recent advances in high-throughput sequencing, multi-omics, and artificial intelligence have accelerated these efforts [5]. In response, major developed countries, including the United States, the United Kingdom, Japan, and South Korea, have launched national-level initiatives on microbiome research and established large-scale human microbiome databases [4,6,7].

These advances via integrated metagenomic and host transcriptomic analyses have allowed for the discovery of various associations between microbial communities and health outcomes [8]. However, most findings remain largely correlative, and the specific role of the microbiome in disease remains elusive [8]. Notably, investigations into gastrointestinal, immune, and neural development require access to gut, immune organ, and brain tissues, which is challenging due to ethical restrictions [9]. Additionally, substantial inter-individual variation in the human microbiome further complicates the identification of consistent microbial signatures and the establishment of universal health markers [10]. Consequently, the difficulty in establishing the microbiome as a causal factor in disease has led researchers to employ human microbiota-associated (HMA) animal models for studying their causal and mechanistic roles in human health [11].

HMA animal models involve germ-free (GF) or microbiota-depleted animals colonized with human-derived microbiota. These models enable human-like microbial communities while minimizing confounders such as environment, diet, and host genetics [11,12,13]. Researchers have applied them to microbial consortia from various human niches, including the gut, oral cavity, and vagina [13,14,15]. However, because the gut microbiome is the most abundant, diverse, and functionally vital microbial community in humans, most HMA studies have targeted intestinal microbiota [16,17]. Gibbons et al. first transplanted human indigenous bacteria into GF mice in 1964 [18]. Since then, HMA rodent models, which are valued for their ease of maintenance, small size, and rapid reproduction, have been widely investigated for host–microbiota cross-talks, with applications spanning immune/inflammatory diseases, metabolic disorders, neurodevelopmental/psychiatric conditions, xenobiotic toxicology, and infectious diseases [19,20]. Nevertheless, their limited translational relevance to humans restricts causal inferences in gut microbiome–disease research [20,21]. To address these limitations, the HMA pig model has been developed, which better recapitulates human gastrointestinal anatomy, immune development, and disease phenotypes [9,22,23,24].

Although prior studies have used HMA pig models, no comprehensive review of their establishment, applications, and future directions has been reported since 2018 [9,25]. This gap highlights the need for an updated synthesis. Hence, this study aimed to conduct a scoping review of HMA pig model establishment methods and fecal microbiota transplantation (FMT) engraftment dynamics. Additionally, we discussed the current research landscape, key limitations, and strategies to enhance model performance.

## 2. Materials and Methods

**Figure 1:**
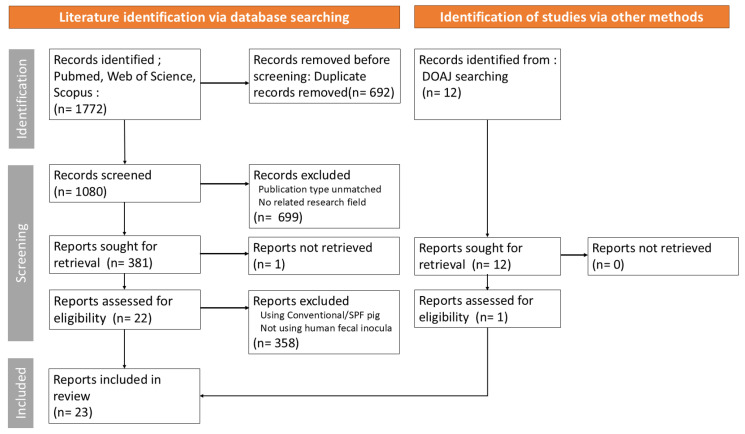
Preferred Reporting Items for Systematic Reviews and Meta-Analyses (PRISMA) flow diagram illustrating the study identification and selection process.

This scoping review aimed to outline the key methodological considerations in establishing HMA pig models as well as to examine FMT engraftment dynamics. This review was guided by the Preferred Reporting Items for Systematic Reviews and Meta-Analyses extension for scoping review (PRISMA-ScR) framework [26]. The PRISMA-ScR checklist and a detailed description of the search strategy are provided in Supplementary Material S1. Literature searches were performed across PubMed, Web of Science, and Scopus, and the Directory of Open Access Journals (DOAJ) website was additionally screened to capture relevant records not indexed in major bibliographic databases from the duration of September 2025 to January 2026. Peer-reviewed studies published during 2007–2025 were included from the time of their initial establishment, with no restrictions on language or publication type. Publications such as reviews, case series, case reports, clinical guidelines, conference abstracts, letters, preprints, notes, and editorials were excluded during the screening stage. In addition, reference lists of relevant systematic reviews were hand-searched to identify any additional eligible studies. The study selection process involved removal of duplicates, followed by screening of titles and abstracts, and then full-text assessment. One reviewer completed all stages of study screening, eligibility assessment, and data extraction without conducting an independent cross-check. The extracted data included study metadata (i.e., title, authors, and year of publication); human donor characteristics (i.e., cohort size, age, health status, and exclusion criteria); sample handling and preparation parameters (i.e., processing materials, collection method, storage medium and conditions, dilution vehicle, dilution concentration, purification approach, and pooling strategy); recipient pig characteristics (i.e., health status, breed, feeding regimen, age at FMT, administration route, dose, and duration); and engraftment assessment parameters (i.e., pig age at observation, collection site, presence of evaluation, assessment method, profiling approach, taxonomic level, and target region). An overview of the study selection process is presented in Figure 1. Charted data were quantitatively analyzed and categorized, and they have been summarized in Table 1 and Supplementary Tables S1–S4.

A total of 1772 records were identified through database searching. After duplicate removal, 699 records were excluded during title and abstract screening due to the lack of relevance or ineligible publication types. Full texts of 381 articles were then assessed for eligibility, of which 359 were excluded because they employed conventional or specific pathogen-free (SPF) pigs instead of GF or antibiotic-induced microbiota depletion (AIMD) models, did not use human fecal microbiota, or relied on defined microbial consortia rather than whole-community transplantation. Consequently, 22 studies were retained from the database searches. Using the same screening procedure, one additional eligible study was identified from the DOAJ database, yielding a total of 23 studies, which were then included in the final analyses.

## 3. Methodological Considerations in HMA Pig Model Establishment

**Figure 2:**
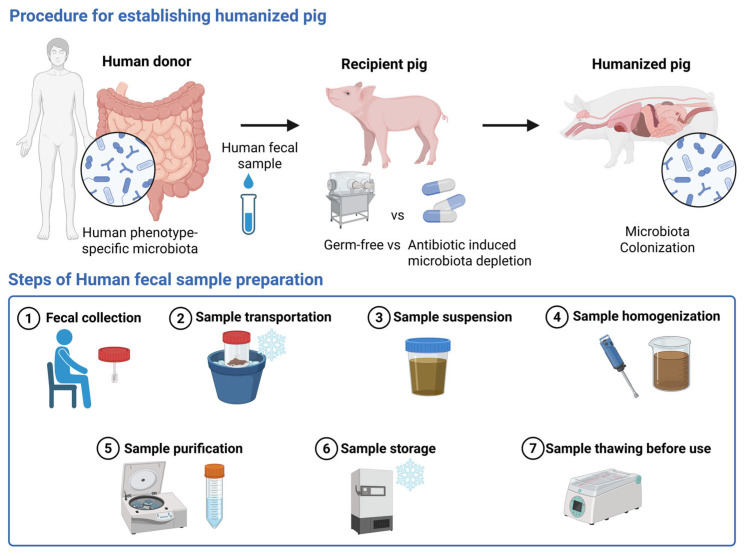
An overview of establishing an HMA pig model. The upper panel illustrates the general workflow. Fecal samples prepared from human donors were transplanted into the Germ-free or AIMD pig model for the generation of HMA pigs. The lower panel depicts the fecal sample preparation process, which consists of seven sequential steps.

In this section, we have presented the methodological landscape of HMA pig studies, including donor selection, sample handling, and FMT preparation protocols, along with recipient pig characteristics. A total of 23 eligible studies, all designed as controlled experimental comparative studies, were included in this section. Figure 2 summarizes the common procedures employed to generate humanized pigs across these studies.

### 3.1. Human Donor Selection

The donor characteristics are summarized in Table 1 and Supplementary Table S1. Across studies, the donor cohort sizes varied (*n* = 1–5), with 69.6% using a single donor [11,22,27,28,29,30,31,32,33,34,35,36,37,38,39,40,41,42,43,44,45,46]. Infant donors are the most commonly used source in HMA pig studies, accounting for 61.5% of studies that utilized infant-derived fecal material. In studies using infant donors, both cesarean- and vaginally delivered infants were included, and most were breastfed; however, exclusion criteria related to the disease status or medication history of infants or their mothers were frequently not reported. Among non-infant human donors, ages ranged from toddlers to older adults, whereas exclusion criteria related to antibiotic use, chronic disease, gastrointestinal disturbance, or medication use were inconsistently reported. Additional criteria included no episodes of constipation, diarrhea, or blood in the stool, and no history of pork consumption outside of designated large animal facilities. Studies that included human donors with specific phenotypes (e.g., obesity or disease) accounted for 21.7%, whereas the remaining studies relied on healthy donors.

In a few studies, GF pigs exhibited marked donor-dependent variation in microbiome following human FMT [29,38]. Owing to substantial donor-related heterogeneity in HMA pig models, it remains challenging to assess the specific effects of donor characteristics on pig gut microbiome outcomes. However, evidence from human FMT studies suggests that such variation is influenced by human age, sex, metabolic status, and lifestyle [47]. The mode of delivery in infants also appears to be an important determinant of gut microbiota diversity and species richness [48].

With reference to the current findings, we propose two recommendations to guide future human donor selection. First, both donor number and selection criteria should be standardized. In other words, including an adequate number of donors is essential to capture inter-individual variation in the gut microbiome and improve the robustness and translational relevance [20].

In addition, the exclusion parameters remain variable across studies, as numerous studies do not report exclusion criteria for donor selection, which possibly compromises reproducibility. A clear definition of donor selection criteria would help reduce methodological heterogeneity [49]. Moreover, enhanced donor screening with a focus on safety is essential. Particular attention needs to be paid to antibiotic resistance genes (ARGs) and zoonotic pathogens in human-derived microbiota. The transplantation of human microbiota into gnotobiotic pigs has been demonstrated to markedly increase the abundance and diversity of ARGs [50]. As ARGs can influence the gut microbiota composition and function [51], their impact on the safety and functional integrity of transplanted microbiota needs to be carefully evaluated in HMA pig models. In addition, zoonotic pathogens such as *Salmonella*, *Shigella*, pathogenic *Escherichia coli*, *Clostridium perfringens*, Rotavirus, *Cryptosporidium*, and *Taenia solium* can infect humans and potentially transmit to pigs through fecal exposure [52,53,54,55,56,57]. As such, rigorous serological and fecal screening of human donors is essential to ensure transplantation safety.

### 3.2. Human Fecal Sample Collection and Handling

Table 1 and Table S2 summarize the parameters related to sample collection and handling. “Frozen” samples were used across all studies, which refers to inoculum prepared from samples that were frozen and subsequently thawed. Only approximately 34.8% (8/23) of the studies reported the sample collection methods. In addition, most studies did not report the sample weight or transport conditions, and only a few noted transportation made on ice or under refrigeration conditions [33,36,38,42,44,45,46]. Fecal sample pooling was performed in 69.6% (16/23) of the studies, which included pooling samples either from different individuals or from different time points of the same donor.

Reconstitution with fresh fecal material resulted in making the gut microbiota compositions more similar to those of donor communities rather than like those obtained using frozen fecal preparations from mice [58]. Therefore, assessing the impact of fresh fecal samples on colonization in pigs may also be informative. Moreover, pooling donor samples should be avoided, as it reduces the effective number of experimental units and increases the risk of pseudo-replication and false-positive results [20]. It is recommended to avoid pooling and instead use an adequate number of donors to capture biological variability in HMA murine models for causal analysis [21]. This principle can also be applied to HMA pig models. In a recent HMA pig study, a randomly selected single-donor inoculum was employed to standardize microbial composition and reduce any inter-individual variability. Notably, mixed-donor inocula could have introduced microbial competition and strain dominance, potentially contributing to unpredictable community structures and the dilution of key microbial signals [46]. This consideration highlights the importance of defining the experimental unit in HMA pig study designs. When a single donor or pooled donor inoculum is used, the donor constitutes the experimental unit, whereas individual pigs represent observational units. Therefore, statistical inference should depend on the number of independent donors or inoculum, rather than on the number of recipient animals [21,59]. To address the lack of methodological detail in past studies, we provided practical guidelines for human fecal sample handling, as informed by commonly used FMT preparation practices. Fresh samples should be collected following natural defecation using sterile, disposable instruments [60]. Sampling weights of more than 50 g are preferred as that improves the transplantation success [60]. Ideally, the samples should be processed within 2 h of collection at best and not later than 6 h. If immediate processing is not feasible, storage at 4 °C or freezing at –20 °C is suitable for short-term preservation, whereas –80 °C cryopreservation is recommended for long-term storage [60,61,62,63,64,65]. Frozen fecal suspensions are practical because they are easy to store; however, direct freezing without cryoprotectants is discouraged [52,66]. Brief oxygen exposure can eliminate nearly half of the microbial population, while prolonged exposure further reduces diversity [67,68]. Therefore, to maximize anaerobe survival and transplantation efficacy, samples should be prepared and handled under strictly oxygen-free conditions at 20–30 °C [52].

### 3.3. Sample Preparation

The key aspects of sample preparation are collated and presented in Table 1 and Table S2. Phosphate-buffered saline (PBS) is mostly used as the suspension medium, often with the supplementation of L-cysteine or pre-reduced to maintain anaerobic conditions and thereby preserve the integrity of the microbial population [69,70]. In contrast, infant formula was used in two studies, whereas one study did not specify the buffer used [12,38,44,45]. The dilution ratios were generally well documented and ranged from 1:10 to 1:50, with 1:20 being the most commonly used ratio. Most of the studies did not report details of the purification step; only five studies mentioned purification, of which four specified centrifugation and one specified filtration. Once prepared, the fecal suspension requires cryopreservation for subsequent use. In most studies, storage at −80 °C with glycerol was used for preservation. Based on the general practices, the preparation methods used to process and store the fecal samples have a significant impact on microbial diversity and viability [60]. Among these, the choice of the suspension buffer plays a critical role. PBS is most commonly used because it maintains a neutral pH throughout the preparation process [71,72], whereas sterile saline (0.9% NaCl) serves as an alternative buffer option [73]. The fecal-to-buffer ratios of 1:3–1:5 are recommended to ensure optimal consistency and microbial viability [74,75]. After dilution, the suspension is typically homogenized and further purified by sedimentation, filtration, or centrifugation to remove debris and reduce viscosity, thereby improving handling and delivery efficiency [49,60]. For application in the model, the prepared fecal suspension must subsequently be cryopreserved. As such, the addition of cryoprotectants before freezing is essential to maintain bacterial cell viability and structural integrity during storage [76]. Glycerol, although unsuitable for lyophilization, is the standard cryoprotectant for liquid cryopreservation and preserves microbial stability for up to six months at −80 °C [60,77]. For short-term storage, direct ultra-low-temperature freezing without cryoprotectants may be adequate [49]. For FMT, samples should be thawed at 37 °C, diluted with sterile saline, and administered promptly at room temperature [52,78].

### 3.4. Recipient Pig Preparation

As an outbred species, pigs more closely mirror the genetic heterogeneity of human populations, making them particularly well suited for modeling human-relevant microbiota–host interactions and recapitulating distinct clinical phenotypes [25]. Methodological parameters related to recipient pigs are summarized in Table 1 and Table S3. In HMA pig models, both purebred pigs (e.g., Yorkshire, Meishan, and Bama) or two- or three-breed crosses are commonly used, most frequently involving Duroc, Yorkshire, and Landrace lineages. To establish pigs for microbiota inoculation, two principal strategies are employed: GF models and AIMD. Among the published studies surveyed, 22 utilized GF pigs, whereas only a single study adopted an AIMD-based approach.

These two approaches elicit markedly distinct host responses, as reflected by distinct patterns of gene expression, physiological outcomes, and behavioral traits, highlighting their fundamental biological divergence [79]. GF pig models are generated through sterile cesarean delivery of pregnant sows, followed by the immediate transfer of newborn piglets into GF isolators to prevent exposure to maternal or environmental microbes [27]. Within these isolators, piglets are maintained under rigorously controlled conditions and provided with sterilized feed, water, and bedding [80]. In most studies, GF piglets are nourished with sterile milk formula, although feeding regimens were not consistently reported (Table S3). Strict aseptic protocols are enforced throughout animal handling, accompanied by routine microbial surveillance and body surface sampling to confirm the maintenance of germ-free status [81].

In contrast, AIMD uses broad-spectrum antibiotics to deplete the gut microbiota theory, providing a facility-independent and convenient alternative [45]. In murine models, individual antibiotics are often used to target manipulation of specific microbial taxa, whereas antibiotic cocktails are employed to achieve more comprehensive depletion of the intestinal microbiota [82]. In one study, 5–6-week-old pigs received daily doses of ampicillin, vancomycin, and neomycin (15 mg/kg each), along with metronidazole (7.5 mg/kg), for 3 weeks; however, the route of administration was not specified. In addition, the effectiveness of intestinal microbial depletion in piglets should be verified following antibiotic treatment, typically by 16S rDNA sequencing [83]. However, the extent of microbiota depletion in pigs following antibiotic treatment was not reported in that study, which may compromise model reliability and reproducibility. Although AIMD approaches are not commonly applied in HMA pig models, murine studies indicate that GF systems allow exclusive human microbiota colonization but are costly, whereas AIMD approaches are more economical yet constrained by residual bacteria [82].

### 3.5. Transplantation Routes and Procedure

FMT suspensions have been administered to animals through multiple routes, including enema, endoscopy, nasogastric tube, and oral delivery [84]. To date, however, oral delivery, via gastric gavage or feed admixture, is the only route reported in HMA pig models. In addition to the delivery route, substantial variability exists in FMT administration protocols, including treatment duration, which ranges from single to repeated inoculations. Inoculation volumes also vary widely, with reported daily doses ranging from 450 μL to 5 mL (Table 1 and Table S3).

In murine models, sodium bicarbonate pre-treatment has been used to neutralize gastric acid and reduce microbial loss during oral FMT administration; however, none of the reviewed pig studies explicitly reported using this approach [85,86]. Comparative evaluation of engraftment outcomes without and with sodium bicarbonate pre-treatment may help determine whether gastric acid neutralization enhances FMT efficacy. This consideration is particularly relevant because certain *Bacteroidetes* species are sensitive to acid-mediated degradation, and gastric passage may substantially reduce their viability during FMT delivery [87]. As an alternative strategy, encapsulated FMT has been shown to improve gut health in weaned piglets [88] and may represent a viable option when oral gavage is impractical.

Overall, this section highlights considerable methodological heterogeneity in the strategies used to establish HMA pig models, underscoring the lack of standardized experimental protocols. The PRIM (Preferred Reporting Items for Microtheraphy) checklist emphasizes transparent reporting of FMT-related parameters to enhance clarity and reproducibility [89]. Accordingly, we recommend that FMT protocols clearly specify the delivery route, inoculation volume, and administration frequency. In Section 6, we propose recommended reporting items for HMA pig models to support standardized protocol development.

## 4. Engraftment Assessment and Compositional Dynamics

Researchers are encouraged to evaluate microbial viability and compositional changes before and after FMT to confirm successful engraftment, using approaches such as culture-based assays, flow cytometry, 16S rRNA gene sequencing, shotgun metagenomics, or agar spot assays [20,60]. In this scoping review, engraftment assessment is defined as the qualitative and quantitative similarity between donor-derived taxa present in the human inoculum and those detected in recipient pigs, restricted to changes attributable to human FMT and excluding effects from other experimental interventions. Of the 23 studies identified in Section 3, 13 were excluded due to the absence of paired pre- and post-FMT microbial profiling. One additional study was excluded because it relied on DNA fingerprinting–based community profiling, which provides limited taxonomic resolution and tends to overestimate microbial similarity [27]. Consequently, only nine studies that assessed engraftment using 16S rRNA gene sequencing were included in the final analysis. These studies exhibited substantial heterogeneity in engraftment assessment methodologies (Supplementary Tables S3 and S4). Given these limitations, this review aims to identify hypothesis-generating trends rather than establish definitive determinants of human microbiota colonization in pigs. The included studies targeted different 16S rRNA hypervariable regions and enabled analyses at various taxonomic levels, including phylum, class, genus, and amplicon sequence variant ASV [12,29,30,33,35,36,38,42,45]. However, explicit criteria or statements defining successful colonization following human FMT were generally lacking, reflecting the absence of standardized benchmarks in HMA pig research. Based on the synthesized evidence (Supplementary Table S4), successful colonization may be operationally defined as the presence of donor-derived taxa in recipient pigs at the sampling time point, with occurrence patterns broadly comparable to those observed in the human donor. Building on this framework, the following section provides a narrative synthesis of the nine studies in which engraftment assessment met PRISMA-ScR criteria.

### 4.1. Early-Phase Engraftment Dynamics

After birth, residual intestinal oxygen in piglets supports the initial expansion of facultative anaerobes, which in turn establish anaerobic conditions conducive to later colonization by obligate anaerobes. Within the first week of milk feeding, these early colonizers are gradually replaced by *Bacteroides*, reflecting a shift toward diet-driven microbial selection [90]. Notably, in the subset of HMA pig studies that reported early post-transplant profiling, comparable successional patterns were observed during the initial engraftment phase, with broadly consistent dynamics within the first week.

In their analysis of early phylum dynamics, Zhang et al. reported the rapid emergence of bacterial communities dominated by *Proteobacteria*, particularly following infant fecal inoculation [29]. These communities subsequently transitioned toward greater diversity, with increasing enrichment of *Bacteroidetes* and *Firmicutes* [29]. Sponseller et al. reported a similar successional pattern in microbial community profiles obtained from days 0–6 after following healthy adult FMT [30]. These shifts are likely driven by pioneer taxa that modify the neonatal gut through oxygen depletion, nutrient reprogramming, and epithelial signaling, thereby creating permissive niches for subsequent colonizers [90,91]. Kumar et al. further demonstrated that this successional pattern is location-dependent [36]. In GF pigs examined 7 days after infant FMT, the small intestine remained dominated by *Proteobacteria* (67–86%) with relatively low levels of *Firmicutes* (13–27%), whereas fecal samples showed more advanced transitions, with *Firmicutes* emerging as the dominant phylum (61%), followed by *Proteobacteria* (37%) [36].

When assessed at finer taxonomic resolution, several studies reported remarkably high similarity between donor and recipient communities. At the genus level, 99.27–100% of operational taxonomic units (OTUs) detected in pig intestinal and fecal samples matched those in the original human inoculum, indicating stable microbial communities by day 7 post-transplantation [36]. Similarly, 10 days after human FMT into 4-day-old GF pigs, 99.94% of recipient fecal OTUs overlapped with the donor inoculum, with only 0.06% unique to the recipients [35]. In another study, 2 weeks after human FMT, colonic outgrowth communities in pigs shared over 95% OTU similarity with the original inoculum [42]. Site-specific analysis revealed that OTUs absent from the original inoculum were relatively more abundant in the duodenum (0.73%) and jejunum (0.66%) than in other intestinal regions, highlighting the influence of local gut environments on community structure. Collectively, these studies suggest a transient dominance of *Proteobacteria*-rich communities in the colon of GF pigs during the first 4–7 days following transplantation. Subsequently, *Bacteroidetes* and *Firmicutes* become established, while donor–recipient OTU similarity remains relatively high during this early phase [29,30,36,38].

### 4.2. Late-Phase Engraftment Dynamics

Longer-term establishment of transplanted microbiota in GF pigs has been evaluated at later time points, typically 4–5 weeks post-transplantation. However, direct quantitative comparisons with original donor profiles are often constrained by reliance on graphical data without explicit numerical values [33,38]. Dhakal et al. analyzed ileal, colonic, and fecal samples and reported that all donor-derived phyla were detectable in feces within four days of transplantation, although pronounced compositional divergence across intestinal sites emerged by 5 weeks [38]. *Bacteroidetes* remained highly abundant in donor inocula as well as in piglet colonic and fecal samples, whereas *Firmicutes* predominated in the ileum. Notably, *Proteobacteria* and *Verrucomicrobia* appeared better adapted to the porcine gut, while *Actinobacteria* were depleted, suggesting selective adaptation of specific phyla within the host environment [38].

Longer-term establishment of transplanted microbiota in GF pigs was evaluated at later time points, typically 4–5 weeks after transplantation. However, precise quantitative comparisons with the original donors were limited by reliance on graphical data without explicit numerical values [33,38]. Dhakal et al. analyzed ileal, colonic, and fecal samples and reported that all donor-derived phyla were detectable in feces within 4 days; however, marked compositional differences across sampling sites emerged by 5 weeks [38]. *Bacteroidetes* were highly abundant in the donor inocula as well as in piglet colonic and fecal samples, whereas *Firmicutes* predominated in the ileum. In addition, *Proteobacteria* and *Verrucomicrobia* were better adapted in piglets, while *Actinobacteria* were depleted, suggesting superior adaptation of certain phyla to the porcine gut environment [38]. In a study by Sponseller et al., *Verrucomicrobia* remained at a consistently low relative abundance (<2%) with minimal fluctuation through day 6 following transplantation [30]. This low-level representation persisted over time, although direct long-term quantitative confirmation was limited. In a study focusing on the large intestinal contents, GF pigs receiving healthy infant FMT initially harbored communities dominated by *Firmicutes* (~90%), which subsequently shifted to a profile characterized by a reduction in *Firmicutes* (~30%) and a corresponding increase in *Proteobacteria* and *Bacteroidetes*. In contrast, piglets with unhealthy infant FMT maintained a higher relative abundance of *Firmicutes* (~70%), whereas *Proteobacteria* increased to ~30% [33,38]. Meanwhile, in AIMD HMA pig models, class-level analysis showed increased *Bacteroidia* and decreased *Bacilli*, indicating a shift in the microbiota toward a human-like profile [45]. Available evidence from related studies suggests that microbiota undergo differential restructuring over time in pigs. The reported patterns vary by intestinal location and donor characteristics, with certain phyla displaying patterns consistent with adaptation to the porcine gut environment.

### 4.3. Consistent Colonization Patterns

To our knowledge, the study by Aluthge et al. is the only published work to have systematically evaluated consistent colonization patterns in HMA pig models [11]. Following transplantation of human microbiota into 6-week-old GF pigs, donor-derived core amplicon sequence variants (ASVs), defined as ASVs consistently present across all aliquots of pooled donor inocula, were monitored at seven time points through day 40 post-transplantation. Among the 27 core ASVs shared by three adult donors, 70–92% were detected at least once in recipient piglets. However, sustained engraftment was more limited, with only 22–74% of these ASVs consistently maintained across four or more sampling time points [11]. At the individual donor level, adult donor microbiota exhibited detection rates of 69–86% (≥1 time point) and persistence rates of 28–57% (≥4 time points), whereas infant donor microbiota exhibited slightly higher prevalence (77–90%) and greater persistence (55–58%). The authors suggested that infant microbiota may achieve more stable engraftment within the porcine gut environment, potentially due to reduced community complexity and greater metabolic adaptability [11].

Notably, persistence alone did not translate into quantitative similarity with the donor microbiota. Among the persistent colonizers, only a limited subset of ASVs maintained abundance levels comparable to those of the corresponding human donors over time [11]. To further illustrate these trends, Supplementary Data 7 and 10 from Aluthge et al. were reanalyzed [11]. Using R software (version 4.5.1), changes in the relative abundance of major piglet taxa at the phylum, family, and genus levels before and after transplantation were visualized. A reproducible workflow and the corresponding code are provided in Supplementary Material S2.

**Figure 3:**
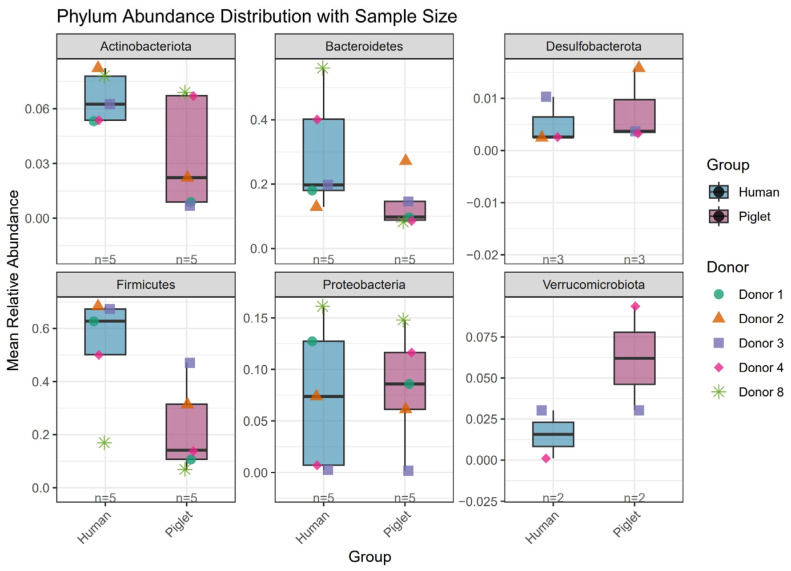
Comparison of the relative abundance of major gut microbiota taxa at the phylum level between human donors and HMA pigs. Box plots were generated from reprocessed data derived from previously published datasets, with the bold central line representing the median. The analyses were based on five human donors; the left and right sides of the X-axis correspond to human donors and HMA piglets, respectively, whereas the Y-axis indicates the mean relative abundance. Sample sizes (*n*) for each of the six major phyla are presented along the bottom of the X-axis and differ by phylum, as only donor samples in which the corresponding phylum was detected were included in the analysis.

Figure 3 characterizes phylum-level dynamics of major donor-derived taxa following transplantation. The analysis revealed a general decline in the relative abundances of *Actinobacteria*, *Bacteroidetes*, and *Firmicutes* after engraftment, while *Proteobacteria* also showed a decreasing trend in most donor–recipient pairs, except for Donor 4. Although not explicitly shown in the figure, the number of core ASVs contributing to each phylum differed between donors and recipients. These observations suggest that, although human donor ASVs can engraft in HMA piglets, donor-like phylum-level abundance patterns are not consistently preserved after transplantation, consistent with observations by Aluthge et al. [11].

**Figure 4:**
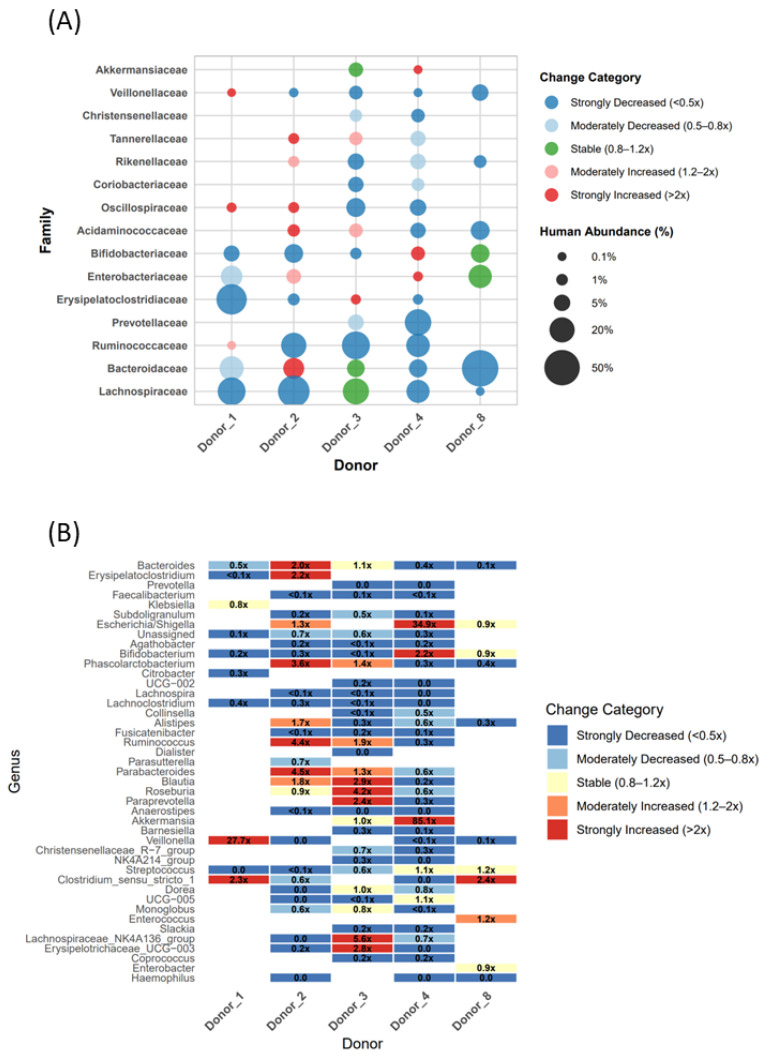
(**A**) Bubble plot illustrating changes in the relative abundance of 15 major bacterial families following FMT from five individual human donors to piglets. The data were generated from reprocessed donor–recipient abundance tables derived from previously published datasets. Bubble size represents the relative abundance of each family in the human donor samples and has been categorized into five bins (0.1%, 1%, 5%, 20%, and 50%). Color indicates the direction and magnitude of abundance change after transplantation, expressed as the piglet-to-human abundance ratio (fold change), and has been classified into five categories, as follows: strongly decreased (<0.5×), moderately decreased (0.5–0.8×), stable (0.8–1.2×), moderately increased (1.2–2×), and strongly increased (>2×). For example, *Bacteroidaceae* accounted for approximately 50% of the total relative abundance in donor_8; however, after transplantation, its relative abundance in piglets decreased to <50% of the corresponding human donor level, indicating a marked reduction after engraftment. (**B**) Heatmap illustrating raw fold-change-based shifts in the relative abundance of 43 major bacterial genera following FMT from the human donors to piglets, based on the same reprocessed datasets. Colors represent the direction and magnitude of change in piglets relative to the corresponding human donors, using the same five-category classification as shown in panel (**A**). Numeric labels overlaid on the heatmap indicate the raw fold change (piglet/human) in the relative abundance. Unlike in panel (**A**), donor relative abundance is not visually encoded in panel (**B**); the heatmap exclusively depicts post-transplantation change states. Blank cells indicate the genera or families that were not detected in the corresponding human donor samples.

Figure 4 showcases the investigation of community-level dynamics by visualizing high-abundance human bacterial families and genera, revealing substantial donor-to-donor variation in the family- and genus-level abundance patterns. This figure visually supports the observations by Aluthge et al. that, despite substantial donor-to-donor variation, *Firmicutes* families, particularly *Lachnospiraceae*, *Ruminococcaceae*, and *Christensenellaceae,* consistently emerge as the key long-term colonizers in HMA piglets [11]. Similar patterns, along with substantial representation of *Bacteroidetes*, were reported by Dhakal et al. [38]. Comparison with past HMA pig studies showed that, despite the differences in the relative abundances, key commensals consistently engrafted across multiple investigations, including short-chain fatty acid (SCFA)-producing genera such as *Bacteroides*, *Faecalibacterium*, *Roseburia*, *Akkermansia*, *Blautia*, *Ruminococcus*, *Subdoligranulum*, *Lachnospira*, and *Phascolarctobacterium*, as well as probiotic genera such as *Bifidobacterium* [11,36,38,42].

Simultaneously, frequent engraftment failure of *Prevotella* and *Dialister* was recorded, along with a marked reduction in the relative abundance of genera such as *Faecalibacterium* and *Lachnospira* in HMA pigs (Figure 4). This pattern reflects the findings of Renu et al., who reported the complete loss of *Prevotella*, *Faecalibacterium*, and *Dialister* from the inoculum in pig colon contents, thereby confirming a shared trajectory across the literature [42]. The authors also observed the emergence of taxa such as *Klebsiella* (1.6%), *Lactococcus* (0.4%), and *Lactobacillus* (0.4%) in outgrowth communities, despite their absence in the original inoculum, further highlighting the selective reshaping of donor microbiota in the porcine host [42]. Altogether, these observations suggest that human-derived microbiota undergo selective reshaping in the porcine gut, with the communities often stabilizing around a restricted set of commensals.

### 4.4. Host- and Recipient-Specific Colonization Patterns

**Figure 5:**
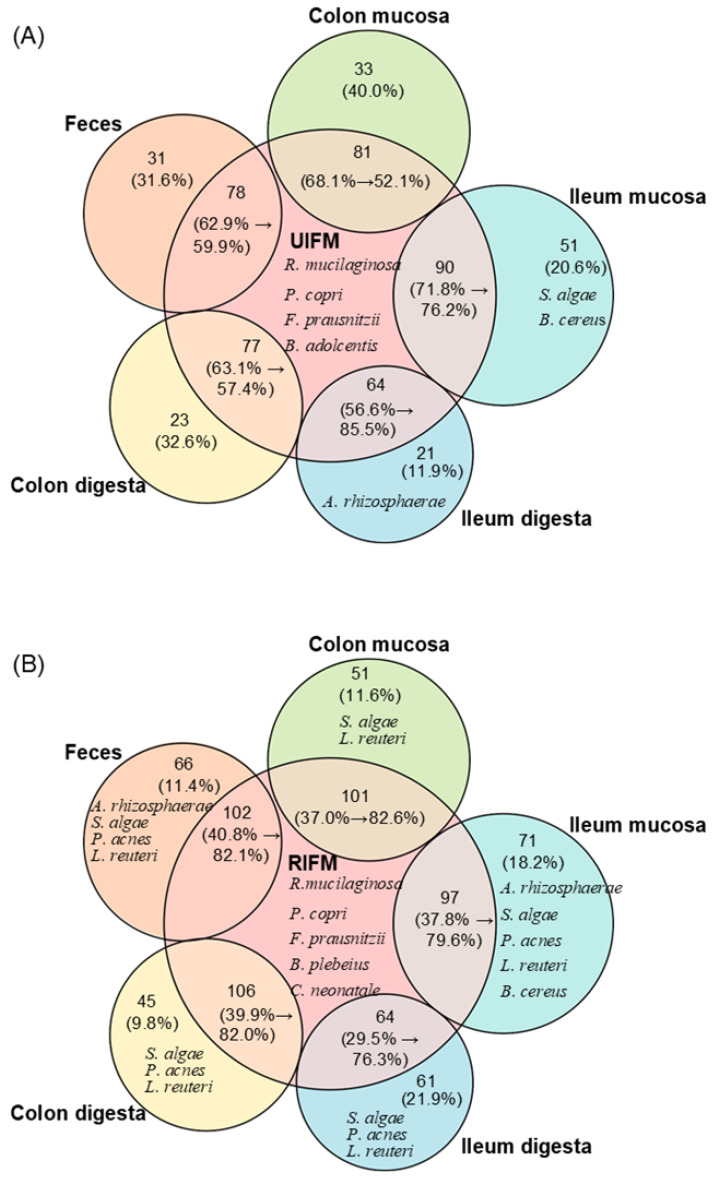
Comparative analysis of bacterial operational taxonomic unit (OTU) distributions between donor infant fecal microbiota and transplanted piglets, based on reprocessed OTU tables from previously published datasets. (**A**) Venn diagram illustrating the overlap of bacterial OTUs between UIFM (urban infant fecal microbiota) and different gastrointestinal sites of UIFM-transplanted piglets. The central circle represents the UIFM donor microbiota, whereas the peripheral circles correspond to different sampling sites: ileum mucosa, ileum digesta, colon mucosa, colon digesta, and feces. The overlapping regions display the number of shared OTUs with relative abundance values recorded in parentheses (donor abundance → post-transplantation abundance). Species-level identification is provided for OTUs unique to either donor or specific tissue sites. (**B**) Corresponding analysis for RIFM(rural infant fecal microbiota) and RIFM-transplanted piglets, following the same visualization scheme as in panel (**A**).

In an HMA pig study by Dhakal et al., OTU- and genus-level analyses revealed complex and stratified patterns of microbial colonization along the porcine gastrointestinal tract [38]. Following FMT into GF piglets, the donor type created distinct engraftment profiles. Significant differences (*p* < 0.05) were detected among the five gastrointestinal sample types, and the majority of OTUs present in the inocula successfully colonized the piglet gut. However, engraftment was not complete, as some taxa emerged only in the piglets, whereas the others present in the inocula failed to establish [38]. However, the analysis did not stratify donor-specific genera by intestinal location, nor did it explicitly report the recipient-specific OTUs. For a more spatially resolved characterization of donor- and recipient-associated taxa, we reanalyzed Supplementary Tables S2b and S3b from Dhakal et al. [38]. The occurrence and taxonomic identities of shared and group-specific OTUs were extracted using R software (version 4.5.1). The resulting data were applied to construct the visualizations presented in Figure 5. A detailed description of the reanalysis, together with the R code and the generated tables, is provided in Supplementary Material S2. Figure 5 summarizes the shared, donor-specific, and recipient-specific taxa across the intestinal regions. Of the inoculated OTUs derived from urban infant fecal microbiota (UIFM; 137 OTUs) and rural infant fecal microbiota (RIFM; 160 OTUs), 46.7–65.7% in UIFM and 40.0–66.2% in RIFM successfully colonized the five gastrointestinal sites. These OTUs accounted for 52.1–85.5% and 76.3–82.6% of the total relative abundance, respectively (Figure 5).

Conversely, transplantation led to the emergence of 21–51 (UIFM) and 45–71 (RIFM) novel OTUs, representing 11.9–40.0% and 9.8–21.9% of relative abundance, respectively. Species-level analysis further highlighted donor- and recipient-specific exclusivity: *Prevotella copri*, *Rothia mucilaginosa*, and *Faecalibacterium prausnitzii* were identified as common donor-exclusive species, with *Bifidobacterium adolescentis* being unique to UIFM and *Clostridium neonatale* and *Bacteroides plebeius* being unique to RIFM. Recipient-specific species included *Aeromonas rhizosphaerae*, *Shewanella algae*, and *Bacillus cereus* in both groups, with *Lactobacillus reuteri* and *Propionibacterium acnes* detected exclusively in RIFM-transplanted piglets. Their intestinal localization within the transplanted piglets was readily discerned across the gastrointestinal regions (Figure 5).

Together, these results were found to be consistent with the inferences of Dhakal et al. [38] that although several donor taxa engraft in HMA pigs, some clinically relevant lineages fail to establish or get selectively lost in the porcine gut. This selective colonization pattern suggested that host-specific factors, including the gut environment and nutrient availability, may influence the composition of transplanted microbiota [38]. This observation highlights the need for the careful selection of HMA pig models when investigating complex human microbiotas, particularly for host-constrained taxa, as also emphasized by Aluthge et al. [11].

### 4.5. Factors Associated with Microbiota Colonization

**Figure 6:**
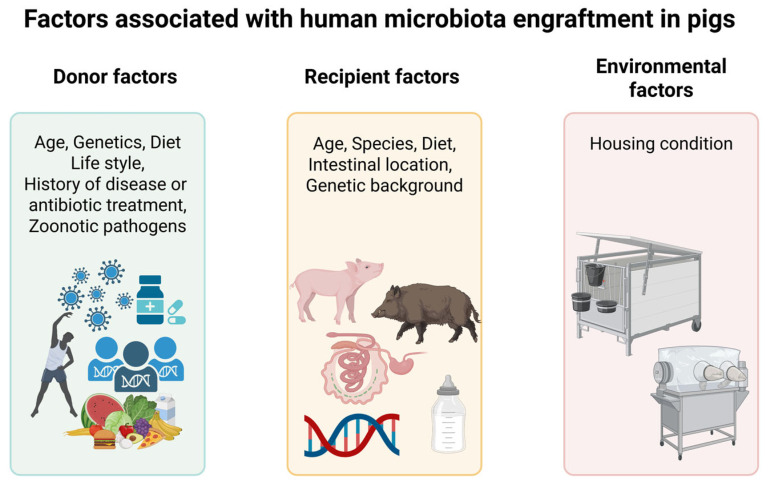
Factors associated with human microbiota engraftment in recipient pigs. The schematic summarizes factors inferred in the studies reporting engraftment-related research.

Herein, we examined FMT engraftment dynamics in HMA pig models. Our analysis of limited evidence revealed that human-derived microbiota undergo selective reshaping in the porcine gut rather than passive maintenance post-transplantation. However, subsequent HMA pig studies have reported conflicting results on microbial stability. For instance, studies have used alpha diversity as a stability indicator but reached opposing conclusions. Zhang et al. observed alpha diversity recovery in infant microbiota and interpreted it as evidence of stability, in contrast to sustained diversity loss in adult microbiota [29]. Conversely, Aluthge et al. cited significant alpha diversity fluctuations in infant-derived communities as evidence of instability, whereas most adult microbiota remained stable and comparable to their sources [11]. These discrepancies in HMA animal models likely stem from three primary factors. First, donor individuality plays a key role, as donor-specific factors such as genetics, diet, and lifestyle strongly influence colonization success [11] (Figure 6). Dhakal et al. supported this by showing distinct microbiota profiles between rural and urban infants, driven by lifestyle-dependent environmental exposures [38]. Second, differences in recipient profiles contribute to divergent outcomes. Aluthge et al. used a uniform cohort of 6-week-old weaned Landrace × Duroc GF pigs at transplantation, whereas Zhang et al. employed GF pigs across a wider age range (5–30 days at inoculation) under varying dietary regimens (milk-fed versus weaned) [11,29]. These studies indicate that recipient age and diet affect microbial colonization. Supported by the FMT principle, breed is another key factor [92]. Moreno et al. emphasized that rigorous control of recipient-intrinsic variables is essential for reproducible and reliable HMA study outcomes [20]. Third, environmental and husbandry conditions influence the microbiota. All HMA pig studies to date, including those above, reared pigs in sterile isolators to minimize environmental impacts. Caroline et al. noted that environmental context shapes microbiota composition and function, potentially leading to divergent phenotypic outcomes across models [93].

### 4.6. Limitations and Translational Considerations

The domestic pig’s anatomical, physiological, and immunological similarity to humans has made it a staple in biomedical and nutritional research [12]. However, even in this favorable host, fully recapitulating a donor’s complete microbial profile remains challenging. In an HMA pig study, human microbiota engraftment rarely exceeded approximately 70% of identified OTUs, showing that complete transmission is difficult even in phylogenetically closer species [38]. At the species level, taxonomic analyses reveal engraftment in HMA pigs is often restricted to a limited subset of taxa (Figure 5). This suggests deterministic microbial colonization patterns, which have also been observed in rodent models [94,95]. Therefore, HMA pig model results warrant cautious extrapolation to humans. These models do not fully replicate complex human clinical symptoms, especially phenotypes absent in pigs. To better mimic human-relevant phenotypes in GF pigs, earlier FMT timing may help, given early-life microbial colonization’s role in host physiological development [96].

## 5. Current Research Status

**Figure 7:**
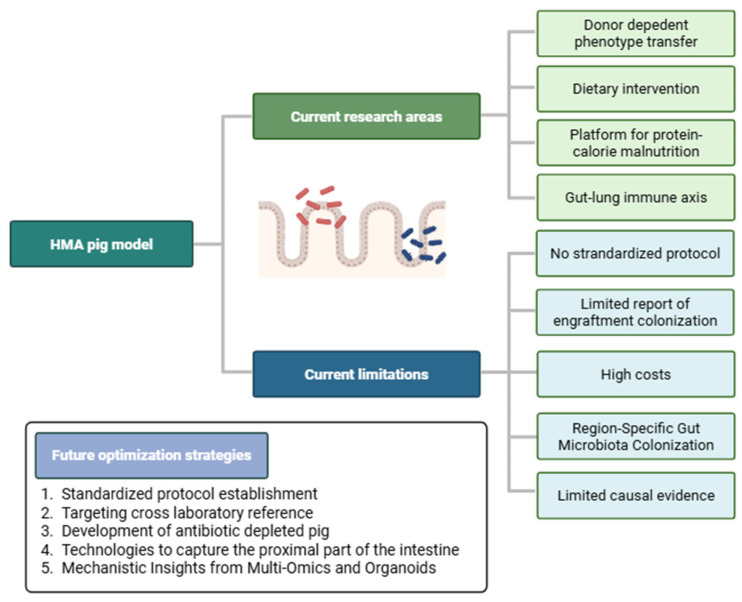
Current Applications, Limitations, and Future Optimization of HMA Pig Models.

The pig model is a powerful alternative to rodents for modeling complex human phenotypes that do not naturally occur in rodents [97]. Similar to humans, piglets exhibit sequential microbial waves during intestinal colonization, with highly comparable functional patterns, underscoring the value of standardized pig models for human gut microbiota research [90]. Since the pioneering work of Pang et al. [27], HMA pig models have evolved from proof-of-concept to structured, mechanistically informative platforms. For instance, Che et al. first examined human FMT effects on gastrointestinal morphology and immune function in GF pigs, reporting that HMA piglets showed increased daily weight gain, jejunal villus height and crypt depth, and counts of goblet cells, IgA-secreting cells, and CD4^+^ T lymphocytes, indicating enhanced mucosal immunity [22]. These results highlight the HMA pig as a valuable in vivo model for host–microbiota–immune interactions. Zhang et al. first applied 16S rRNA sequencing to assess colonization by adult or infant human fecal microbiota in GF pigs, targeting the ~60-nucleotide V6 hypervariable region of the 16S rRNA gene to generate ~200 nt amplicons on the Illumina HiSeq2000 platform [29]. The short region’s limited phylogenetic resolution restricted taxonomy to the phylum level [29]. More recently, Aluthge et al. performed longitudinal ASV-level analysis of human FMT in GF pigs using donors with varying microbial diversity, and Zhang et al. used integrated multi-omics to reveal human-like remodeling of serum metabolomes and immune cell transcriptional programs in HMA pigs [11,45]. This section summarizes recent advances in HMA pig models, including interventions and disease-associated microbial shifts (Figure 7).

### 5.1. Donor Dependent Phenotype Transfer

Many studies show that environment- or disease-associated human gut microbiota alterations transfer functionally to HMA pig models, yielding distinct immune, metabolic, and growth phenotypes. Dhakal et al. compared rural and urban infant microbiota, demonstrating that early-life microbial ecology differences deterministically shape host physiology and immune development [38]. Similarly, Amimo et al. investigated the ability of the gut microbiota from stunted children to functionally reproduce growth and metabolic abnormalities [46]. Twitchell et al. compared alpha- and beta-diversities between healthy and unhealthy human gut microbiota, confirming distinct communities [33]. Distinct donor communities establish reproducible taxonomic profiles in recipient piglets, which, in turn, shape host physiological processes and gene expression programs [38,46]. Importantly, these microbial and metabolic differences result in distinct mucosal immune outcomes, as evidenced by the increased conventional dendritic cells (DCs) and reduced helper T cells (Th cells) and monocytes in pigs colonized with Amish infant microbiota, indicating a skewed mucosal immune profile. Correlation analyses have also linked taxa such as *Clostridium* and *Bacteroides* to these immune shifts, further supporting a donor-dependent imprint on mucosal immunity [38]. Twitchell et al. further reported the presence of distinct vaccine-induced immune responses, with Healthy human gut microbiota (HHGM) enhancing T-cell and mucosal antibody responses and reducing viral shedding, and Unhealthy human gut microbiota (UHGM) leading to weaker immunity and increased viral replication [33]. Human rotavirus (HRV) infection was found to markedly reshape the HHGM, but it only caused minor changes in UHGM pigs, consistent with their pre-existing dysbiosis [33]. *Turicibacter*, detected only in the HHGM group, may be implicated in the immunomodulation of HRV severity [25,35,36]. Moreover, transplantation of host-specific microbiota into secondary GF recipients has been shown to consistently reproduce the growth-retarding phenotype, confirming its transferability and causality [46]. Collectively, these studies indicate that donor-specific human gut microbiota engraft in HMA pigs and drive distinct immune, metabolic, growth, and infection-related phenotypes.

### 5.2. Dietary Intervention

GF pig and mouse models are widely used to evaluate pro- and prebiotics, but their translational relevance is limited by the absence of an intestinal microbiota or its divergence from the human gut microbiome [25]. To address these limitations, HMA pig models offer superior physiological relevance for translational microbiome research. Recent studies have leveraged this platform to examine how dietary interventions modulate human gut microbiota responses to HRV infection during early life.

*Escherichia coli* Nissle 1917 (EcN), a Gram-negative probiotic commonly used to treat ulcerative colitis, establishes persistent colonization in the gut microbiota [98,99]. Michael et al. showed that delivering EcN as a biofilm on dextranomer microspheres reduced diarrhea and viral shedding, enhanced mucosal and systemic immunity, and upregulated genes involved in epithelial repair and barrier function [41]. Notably, these findings demonstrate that the delivery system enhances probiotic persistence and functional efficacy in the gut [41]. Follow-up studies have revealed that co-administering EcN with tryptophan synergistically improves clinical outcomes by reducing diarrhea, promoting weight gain, and increasing survival. These benefits stem from restored intestinal absorptive function and homeostasis, along with enhanced immune and anti-inflammatory responses, normalized tryptophan metabolism, and increased production of immunoregulatory metabolites. Improvements in lipid and nucleotide metabolism further support immune function, energy balance, and growth in malnourished hosts, positioning this combination as a promising prophylactic strategy against HRV infection in vulnerable populations [43].

Another research focus involves *Lactobacillus rhamnosus* GG (LGG), a well-studied Gram-positive probiotic that enhances intestinal barrier integrity and supports gut health [100]. LGG has been extensively evaluated in clinical trials for preventing or shortening rotavirus-associated diarrhea in children [101,102]. Building on these findings, HMA pig models have dissected LGG–microbiota–immunity interactions, providing deeper insights into its effects during HRV infection. A study by Zhang et al. showed that HRV challenge induced a phylum-level shift from *Firmicutes* to *Proteobacteria*; LGG supplementation prevented this dysbiosis by stabilizing core taxa, highlighting the model’s value for studying host–microbe–virus–probiotic interactions [32]. Despite these microbiota-stabilizing effects, LGG did not significantly reduce diarrhea severity or viral shedding, indicating limited enhancement of HRV vaccine efficacy in that context [32]. In a follow-up study, LGG supplementation in HRV-vaccinated HMA piglets boosted innate immunity and promoted interferon (IFN)-γ–producing T cells and Th1-type responses in a dose-dependent manner, though these changes did not increase antibody production [31,34].

Meanwhile, Shen et al. used the HMA pig model to assess fructo-oligosaccharides, a well-established prebiotic fiber [28]. Supplementation consistently promoted bifidobacterial expansion and induced dynamic shifts in non-bifidobacterial taxa, including *Bacteroides* and *Clostridium* subgroups, across developmental stages. However, without species-level resolution or mechanistic studies of host–microbe interactions, these findings remain primarily correlative [28]. Sponseller et al. took a different approach, evaluating the therapeutic potential of hyperimmune bovine colostrum (HBC) in HMA pigs, rather than traditional pre- or probiotic interventions [30]. This study did not examine the functional role of the humanized microbiota, focusing instead on HBC efficacy under humanized gut conditions. They showed that *Clostridioides difficile* toxin-specific HBC effectively combats *C. difficile* infection without disrupting the normal human gut microbiota. Given the human-like gastrointestinal phenotypes induced by *C. difficile* in pigs, HMA models provide a clinically relevant platform for testing targeted interventions [30]. Together, evidence from these studies confirms that HMA pig models offer a physiologically relevant platform for evaluating dietary interventions and their interactions with the gut microbiota, immune responses, metabolism, and disease outcomes.

### 5.3. Mechanistic Insights into Protein-Calorie Malnutrition (PCM)

Pig studies have substantially advanced our understanding of how malnutrition affects growth, gut physiology, and multi-organ development [103]. Researchers at The Ohio State University’s Food Animal Health Research Program used the HMA pig model to recapitulate key features of PCM under a protein-deficient diet and to delineate underlying immune–metabolic mechanisms [25,35,36,37,40,104]. Collectively, these studies establish HMA pig models as a robust translational platform for elucidating interactions among nutrition, the gut microbiota, and immune function [25].

PCM symptoms were more pronounced in HMA piglets than in GF counterparts, coinciding with reduced microbial diversity and time-dependent declines in *Bacteroidetes* [25,35,37]. Mechanistically, protein deficiency in HMA pigs sharply reduced tryptophan availability, reprogramming the tryptophan–kynurenine metabolic pathway [37,40]. This nutrient stress triggered broad immunosuppression, including decreased frequencies and/or function of natural killer (NK) cells, plasmacytoid DCs, CD103^+^ DCs, and apoptotic mononuclear cells [35]. In parallel, it activated General Control Nonderepressible 2, a nutrient-sensing kinase that suppresses immune signaling and T cell proliferation, along with coinciding with increased global DNA methylation in the intestinal epithelium, potentially silencing genes essential for immune responses and epithelial regeneration [25].

At the immunological level, protein deficiency disrupted the DCs–interleukin (IL)-12–NK innate immune axis, reducing production of IFN-α, IL-12, and tumor necrosis factor (TNF)-α, as well as frequencies of CD103^+^ DCs and NK cells. This innate impairment coincided with dysregulation of the IFN-γ/indoleamine 2,3-dioxygenase 1 (IDO1) signaling pathway, including fewer IFN-γ-producing T cells, increased regulatory T cells (Foxp3^+^), and suppressed IDO1 expression [37,40]. Fischer et al. reported consistent reductions in CD4^+^ T cells, CD8^+^ cytotoxic T cells, Toll-like receptor (TLR) signaling, and antigen-presenting cell populations, all contributing to weakened antiviral immunity [104]. At the mucosal interface, protein malnutrition lowered intestinal and serum levels of angiotensin-converting enzyme 2, potentially compromising mucosal immunity by impairing tryptophan uptake and epithelial barrier function [104]. However, many of these mechanisms rely on correlational data and require further validation to establish causality.

Protein malnutrition markedly reduced expression of key epithelial genes, including villin, Mucin 2, Chromogranin A, SRY-Box Transcription Factor 9, Proliferating Cell Nuclear Antigen, in the ileum and duodenum, signaling compromised epithelial integrity, mucus production, and regenerative capacity. This suppression reflected broader structural damage to the intestinal barrier and impaired mucosal healing, as shown by elevated IFN-γ, TNF-α, TLR2^+^ immune cells, and serum endotoxin levels [37]. Such loss of mucosal integrity promotes bacterial translocation into systemic circulation, perpetuating immune dysfunction and susceptibility to enteric viral infections [25,35]. These immune impairments worsened HRV outcomes, with elevated viral titers indicating compromised adaptive immunity [104]. HMA pigs on a protein-deficient diet showed reduced weight gain, more severe diarrhea, and lower HRV vaccine efficiency, with effects being even more pronounced in GF pigs, underscoring the microbiota’s partial protective role [33,40]. These findings suggest that PCM-associated gut microbiota dysbiosis harms host health and metabolic outcomes, possibly via impaired energy extraction, nutrient assimilation, microbial competition for host nutrients, or production of deleterious metabolites from altered microbial activity [25]. Overall, HMA pig studies position this model as a translationally relevant platform for exploring PCM-related interactions.

### 5.4. Gut–Lung–Immune Axis in Influenza A Virus (IAV)

Pigs serve as natural influenza hosts with anatomical, immunological, physiological, and genetic similarities to humans, making them more suitable than rodents for studying influenza pathogenesis and host responses [9,105,106]. Recent studies underscore the gut microbiota’s pivotal role in shaping innate and adaptive immune responses to IAV, promoting early viral clearance and limiting inflammation-mediated tissue damage via the gut–lung axis [107]. HMA pig models infected with IAV have provided key insights into this gut–lung–immune axis [42,44]. Following IAV infection, gut microbiota undergoes phylum-level shifts in a microbiota-dependent manner, including increases in potentially pathogenic genera such as *Klebsiella* and *Clostridium*, which may elevate secondary gut infection risk. Furthermore, specific gut bacteria, including *Turicibacter*, *Klebsiella*, *Akkermansia*, and *Lactococcus*, show strong correlations in relative abundance between intestinal and respiratory tissues [42,44]. Beyond composition, microbiota alterations modulate lung immune cell profiles, cytokine expression, and systemic antiviral responses, particularly via SCFAs and type I IFN signaling [42,44]. Notable correlations have linked bacterial genera to host immune parameters; for example, *Bifidobacterium* abundance negatively associates with IL-4 expression and myeloid cell counts, whereas *Klebsiella* inversely correlates with IL-4 levels and cytotoxic T cells. Longer colonization (≥5 weeks) is required to establish microbial maturity and to induce immune phenotypes. Renu et al. reported the critical roles of both gut microbial composition and colonization duration in shaping microbial communities in the respiratory tract and systemic immune compartments, supporting the concept of a functional gut–lung–immune axis [42].

In the context of obesity, Renu et al. showed that pigs colonized with healthy human fecal microbiota exhibited stronger immune cell activation, lower pro-inflammatory cytokines (e.g., IL-6, IL-12, TNF-α), higher SCFA-producing bacteria, and reduced pro-inflammatory taxa compared to those with obese human fecal microbiota (oHFM) after IAV infection [42]. Although respiratory viral loads were similar, oHFM pigs displayed heightened inflammatory responses, suggesting that oHFM predisposes the host to exaggerated respiratory inflammation during IAV infection [42]. Together, these findings highlight HMA pig models’ utility for mechanistic studies of the gut–lung–immune axis and its role in antiviral immunity.

## 6. Current Limitations and Strategies for Improvement

Wang et al. were the first to comprehensively review the HMA pig model and highlighted its utility for examining how environmental factors affect gut microbiota and gastrointestinal, immune, and neural development [9]. They further noted that metabolomics and metaproteomics in this model could identify potential biomarkers for disorders such as asthma, eczema, necrotizing enterocolitis, inflammatory bowel diseases, obesity, and autism, while clarifying the microbial mechanisms underlying disease pathogenesis [9]. However, the subsequent research on HMA pig models has remained largely focused on microbiome–host interactions in the context of PCM and on evaluating specific dietary interventions during the next decade, with studies incorporating metabolomics or proteomics approaches remaining limited [43]. Consequently, most disease domains originally proposed by Wang et al. [9] remain unexplored. This contrasts sharply with the HMA murine model, which has been widely employed across multiple biomedical fields, including oncology, metabolic disorders, diabetes, cardiovascular health, and neurodevelopment, utilizing omics methodologies such as metabolomics and proteomics [108,109,110,111,112,113]. Notably, it has been extensively used to investigate the gut–brain axis and the microbiota’s influence on neuropsychiatric and behavioral conditions, such as anxiety, depression, schizophrenia, and autism spectrum disorder [85,114,115,116,117]. This section discusses the key limitations and translational barriers of the HMA pig model, along with strategies to improve its translational utility (Figure 7).

### 6.1. Technical Limitations and Optimization Strategies

Despite various advances, the HMA pig model methodology still requires systematic refinement, including the optimization of transplantation protocols. The lack of methodological uniformity across research groups presents a major challenge [49]. For instance, protocols for human donor selection, fecal sample collection, and transport often differ among studies or are insufficiently documented (Table 1, Tables S1 and S2). Future studies need to focus on minimizing variability arising from donors’ physiological, lifestyle, and dietary differences, which considerably contribute to heterogeneity in HMA pig model outcomes. Moreover, substantial variation exists in core experimental parameters, including the dose and duration of fecal administration and the age of GF pigs at transplantation (Table 1 and Table S3). Collectively, these differences underscore the lack of a standardized protocol for HMA pig research, which hinders the reproducibility of study outcomes. To address these challenges, consistent reporting of the fundamental methodological elements is warranted when establishing HMA pig models.

Table 2 provides a minimal reporting framework informed by the evidence discussed in this review to support the development of standardized protocols. The structured reporting is expected to improve study comparability and to facilitate more robust meta-analyses and systematic reviews of HMA pig research. Meanwhile, this field would greatly benefit from the development of a cross-laboratory reference microbiota, analogous to the defined and stable microbial communities used in HMA mouse models, such as the Altered Schaedler Flora (ASF) and Oligo-Mouse-Microbiota (Oligo-MM12). Therefore, establishing a more standardized inoculum for HMA pigs can help improve reproducibility and address the current limitations related to protocol variability.

Additionally, the failure to report engraftment efficiency further undermines the reliability of findings. Even among studies that do report it, the field lacks a standardized, quantitative benchmark for success. This necessitates the establishment of specific criteria, such as the percentage similarity of the human donor microbiota at finer taxonomic resolutions (e.g., OTU or ASV level) in recipient pigs, for validating the model’s fidelity and enabling cross-study comparisons [11]. Notably, Seedorf et al. showed that dietary niche pressures are the predominant host-derived force shaping gut microbiota assembly in the HMA mouse model [94]. Microbial capacities for carbohydrate and bile acid metabolism largely determine colonization success, whereas adaptive immunity and gastric acid play relatively minor roles [94]. Therefore, it is necessary to determine if similar phenomena can be reproduced in GF pigs by modulating dietary composition and the bile acid milieu, while accounting for donor variability, recipient factors, and delivery routes. Accordingly, future studies on engraftment efficiency should encompass taxonomic engraftment across different gut regions and associated physiological and immunological responses, along with functional stability indices such as SCFA profiles, bile acid conversion patterns, and the abundance of key functional genes. In this context, genome-centric metagenomics facilitates identifying colonizing taxa and their functional potential, and integrated multi-omics approaches allow systematic evaluation of maintenance of human-like functions in the transplanted pig gut ecosystem.

Maintaining gnotobiotic pig facilities entails substantial costs and technical complexity, requiring highly trained specialized personnel. A single GF pig litter costs roughly $25,000, about 50 times more than GF mice, and daily housing fees for conventional pigs can reach $19, compared with $1–3 for rodents. The limited availability of large-animal facilities further exacerbates financial and logistical burdens [118]. These high costs and logistical complexities commonly constrain donor cohort sizes in HMA piglet studies [11,22,27,28,31,32,35,36,37,39,40,41,43,104]. To mitigate these constraints, AIMD in pigs may serve as a practical alternative to GF pigs for certain experiments. Although residual or environmental microbes in non-gnotobiotic settings may hinder human microbiota engraftment, AIMD remains a promising, cost-effective alternative [45,119]. However, its application in the HMA pig model remains limited and underexplored. To address this, future studies need to compare gnotobiotic approaches, particularly transplantation outcomes and long-term microbial stability in GF versus AIMD pigs. Mouse studies support this approach, showing that AIMD-SPF mice exhibit better early engraftment, whereas colonized GF mice retain donor-like communities and display more persistent intestinal functional changes [86]. Such comparisons are essential for selecting optimal pig models for human FMT, and broader adoption will require standardizing antibiotic regimens across compositions, doses, durations, and routes [119,120].

Finally, fecal samples capture only a distal colonic snapshot of the gut ecosystem. Analyses across 14 intestinal sites in genetically homogeneous pigs have revealed that fecal microbiota and metabolites fail to reflect the distinct profiles in the stomach, small intestine, or proximal colon [121]. Consistent with this site-specific pattern, HMA pig studies report that the relative abundance of major bacterial phyla in feces mirrors the colon but not the ileum, regardless of donor source [38]. Moreover, a study observed close phylogenetic resemblance between fecal and colonic communities, supporting the use of non-invasively collected fecal samples to represent colonic composition, while still failing to capture features present in proximal gut regions [29]. Emerging capsule-based technologies, orally administered to pigs, enable non-invasive, colon-specific activation and recovery of microbial communities that closely resemble the native colonic environment. These represent a promising approach for precise, region-targeted sampling [122]. Additionally, the cecum-cannulated HMA pig model offers a practical platform for longitudinal, site-specific sampling to comprehensively assess gut ecological dynamics [123].

### 6.2. Key Considerations for Enhanced Translation of the HMA Pig Model

Regarding the advantages of pigs as translational animal models, pigs are omnivorous like humans [124], and they share key physiological and anatomical features, including the gastrointestinal tract and colon-fermenting digestive system [125]. Furthermore, pigs exhibit an immune system similarity of >80% with humans, along with up to 95% genomic and proteomic homology [25]. Their extended susceptibility to human pathogens and ability to replicate human clinical phenotypes further support their value as a robust translational model [35,126,127,128,129,130,131]. Notably, approximately 96% of their functional pathways overlap with the human gut microbiome, and they present a highly similar gene profile for secondary bile acid production. These advantages position pigs as a particularly suitable model for investigating metabolic processes in humans [132,133].

Many studies have investigated physiological crosstalk between the gut microbiome and distant organs using conventional and GF pig models. Once established in the porcine gut, the microbiota supports host homeostasis through direct epithelial interactions and microbial metabolite production [134]. Gut microbiota colonization induces transcriptomic reprogramming in the hypothalamus, supporting the existence of a gut–brain axis [135]. Additionally, probiotic administration improves gut microbial composition while attenuating pulmonary immune and inflammatory responses via the gut–lung axis [136]. For instance, in a chronic kidney disease-induced minipig model, modulation of specific intestinal bacterial genera reduced uremic toxin levels and ameliorated renal dysfunction, providing direct evidence for gut–kidney crosstalk [137]. Moreover, correcting the gut–heart inflammatory axis in a cardiometabolic disease model significantly improved pathological structural remodeling in the left atrium and ventricle, while recent studies further highlight the gut–muscle axis by linking microbial and metabolic signatures to skeletal muscle development [138,139]. Although studies using HMA pig models for gut–organ axes remain limited, their strong physiological relevance to human metabolic diseases positions them for more in-depth, translational investigations in future research [140,141].

A previous review highlighted the pig’s superior translational value among HMA animal models [13]. However, using pigs as translational models introduces systematic biases. Ecological determinants of human disease, such as diet, geographic context, and lifestyle, are challenging to fully recapitulate in HMA pig models. Moreover, environmental factors (e.g., sanitary conditions) and behavioral traits (e.g., coprophagy) can confound the pig gut microbiota, contributing to translational limitations from inter-genus differences in microbiota [25,125]. Species-specific differences in Peyer’s patches may also influence discrimination between pathogenic and commensal bacteria, particularly in pig-based models of the human intestine [142], warranting further investigation. Additionally, pigs lack transplacental antibody transfer; HMA piglets are immunologically naïve at birth and may not fully replicate the passive immunity in human neonates, potentially limiting the accuracy of vaccine response studies [143,144]. These limitations complicate the direct translation of pig-based findings to human clinical applications. Table 3 presents representative examples of appropriate and cautionary uses of the HMA pig model. These systematic biases introduced by the pig model need to be carefully considered when designing future HMA pig-based studies to enhance translational relevance.

Among these research areas, the gut–brain axis stands out as relatively underexplored and highly promising for future investigation. A growing body of research has examined the human microbiome’s influence on neurological disorders [145,146,147], where gut microbiome dysbiosis is commonly reported [148,149,150]. FMT in these disorders has been associated with improvements in symptoms and inflammation. However, evidence remains limited and heterogeneous, underscoring the need for standardized, large-scale, double-blind randomized controlled trials [151,152]. Proposed mechanisms include attenuation of neuroinflammation, restoration of gut barrier integrity, and modulation of microbiota–gut–brain axis signaling via microbial metabolites (e.g., SCFAs), gut hormones, and vagal pathways [152]. Reportedly, pigs share human-like brain developmental trajectories and gyrencephalic brain morphology, enabling the use of human clinical neuroimaging protocols and spatial learning tasks such as the T-maze [153]. This positions the HMA pig model as a valuable platform for translational studies of these mechanisms.

**Table 3:** Representative best-fit and cautionary translational use cases of the HMA pig model, with supporting evidence.

Supporting Evidence	Best Fit Use Cases	Cautionary Use Cases
Polling increases the risk of pseudo-replication and false-positive results [20]	Use of a sufficient number of donors without pooling	Use of a single donor as a representative phenotype or pooled samples
A clear definition of donor selection criteria would help reduce methodological heterogeneity [49]	The study applied standardized donor selection and exclusion criteria (e.g., ARGs and pathogen screened)	The study does not apply the donor exclusion criteria (e.g., no serological and fecal screening)
Sample handling time and storage conditions influence microbial composition and diversity [60]	The study processed samples within 2 h of collection under oxygen-free conditions at 20–30 °C.	The study processed samples ≥6 h after collection without refrigeration or freezing.
Dietary changes can induce stress in pigs and alter the gut microbiome [154]	The study maintained a single-diet feeding regimen (e.g., sterile milk) until the time of sampling.	The study implemented a dietary transition before sampling (e.g., from sterile milk to solid feed).
Failure to verify depletion may compromise reproducibility.	The study examined the efficacy of intestinal microbial depletion after AIMD.	The study did not assess the efficacy of intestinal microbial depletion after AIMD
Animal models are more appropriate for comparative, directionality-based inference than for direct translation to humans [155]	The study evaluated whether microbiota from distinct human populations induce distinguishable immune, metabolic, and growth phenotypes in pigs.	The study performed preservation of specific donor taxa as the primary criterion for transplantation success.
Emphasis should be placed on the relative differences, directionality, and pattern preservation rather than absolute replication.	The study evaluated whether relative patterns or functional outcomes associated with different donor microbiotas are preserved after transplantation.	The study directly extrapolated the clinical protective or risk effects of specific microbes from pig models to humans.
In pig models, partial engraftment should be acknowledged.	The study examined how selectively engrafted microbial communities in the pig gut influence host physiology.	The study assumed complete reconstruction of the donor microbiota in recipient pigs.
When thousands of OTUs are assessed for association with a limited number of conditions, many apparent taxon-level associations are expected to arise by chance alone [156].	The study tested the causal relationship between transplanted microbiota and host phenotypes.	The study sought to establish direct causal roles for individual microbial taxa.
The ability of animal models to reproduce human diseases at a quantitative or diagnostic level is limited.	The study investigated functional mechanisms underlying the gut–lung–immune axis.	The study aimed to achieve quantitative or diagnostic-level replication of human clinical diseases.

### 6.3. Research Gaps and Future Direction

A major research gap in HMA pig models is the lack of mechanistic studies establishing causal relationships. In HMA rodent studies, many findings are correlative, offering associative rather than mechanistic links between microbiome alterations and human disease-related phenotypes [32,33,36]. Walter et al. critically argued that HMA rodent studies often overstate causal claims, citing an implausibly high success rate (approximately 95%) for phenotype transfer; they caution that this undermines microbiome science credibility and call for rigorous causality inference [21]. Similarly, most HMA pig studies focus on how dietary, probiotic, pathogen, or vaccine interventions affect microbiota composition, host metabolism, immunity, or infection outcomes. Despite providing improved mechanistic insights, these findings remain largely correlative and have yet to elucidate specific molecular and cellular pathways. It remains unclear how defined microbial taxa or their metabolites causally drive human-relevant phenotypes or disease outcomes. Accordingly, future research should prioritize elucidating these host–microbiota interaction pathways and evaluating therapeutic approaches.

Another limitation of HMA pig research is the prevalence of single-instance studies without follow-up, likely due to the substantial costs outlined above. For instance, human FMT has been shown to enhance human norovirus infection in GF pigs, accompanied by shifts in gut microbial composition, particularly increased *Proteobacteria* and *Firmicutes* [39]. These findings suggested that specific human microbiota components facilitate viral replication and pathogenesis. However, the authors noted potential donor- or strain-specific effects but did not validate generalizability using diverse fecal microbiota or multiple human norovirus isolates. Similarly, in 2008, Che et al. reported that HMA pigs exhibited changes in intestinal epithelial development and mucosal immune cell profiles [22]. Yet key mechanistic questions remained unanswered: the precise microbial factors driving epithelial proliferation and differentiation; specific antigens or metabolites enhancing immune cell activation; and bacterial stimuli increasing major histocompatibility complex class II expression. The authors explicitly highlighted these as priorities for future investigation [22]; however, as with many HMA pig studies, no follow-up research has been conducted.

Complementary use of human intestinal organoid models alongside HMA pig models holds promise for addressing these limitations through mechanistic validation. Combined with advanced multi-omics approaches, human organoids provide high-resolution insights into cellular and molecular mechanisms. However, they are limited by challenges in incorporating complex microbiota and by their inability to capture systemic immunity or whole-organ physiology [157]. Parallelly, in vitro fermentation models offer a host-independent system for exploring interactions within complex human gut microbial communities [17]. Strategically integrating human organoids and in vitro fermentation systems with HMA pig models could overcome the limitations of each model, leveraging the mechanistic precision of in vitro platforms and the physiological relevance of in vivo pig models to enhance both interpretability and translational potential.

### 6.4. Limitations of the Scoping Review

This study has some limitations. First, a single reviewer conducted all stages of the review process; the absence of an independent duplicate review may have introduced selection or data extraction bias. Second, the lack of published studies on human FMT in GF pigs, together with the overall weakness of available evidence, may have restricted our ability to fully contextualize reported engraftment efficiency. Despite these limitations, this scoping review is the first to systematically synthesize the present literature, outlining key methodological considerations for establishing HMA pig models and examining FMT engraftment dynamics.

## 7. Conclusions

In this scoping review, we systematically synthesized methodological approaches used in HMA pig studies, covering human donor selection, FMT procedures, and post-transplant engraftment assessment. This revealed substantial, unstandardized heterogeneity across study designs. Although the limited number of studies preclude broad generalizations, the available evidence suggests that human-derived microbiota undergo selective reshaping in the porcine gut. Microbial communities often stabilize around a restricted set of commensal taxa, with compositions varying by intestinal location. Colonization patterns were also influenced by human donor characteristics, recipient pig factors, and housing conditions. The HMA pig model offers many distinctive advantages, including genetic uniformity, amenability to colonization by human microbiota, and substantial overlap in functional metabolic pathways between humans and pigs, despite various technical limitations and its relatively limited adoption to date. These challenges can be addressed through systematic refinement of experimental strategies across four major domains. First, standardizing technical protocols is essential to improve colonization efficiency and inter-study reproducibility. Second, establishing a standardized inoculum would enhance reproducibility and reduce protocol variability. Third, researchers should carefully evaluate GF versus AIMD pigs based on study duration and cost, applying these models selectively according to specific experimental objectives. Fourth, integrating location-specific sampling approaches would enable more systematic assessment of organ-wide microbiota impacts, thereby strengthening the model’s physiological and translational relevance. Finally, when combined with human organoid systems, in vitro fermentation platforms, and multi-omics approaches, the HMA pig model promises a powerful framework for rigorously delineating causal relationships and mechanistic pathways linking the human microbiome to host physiological responses. Altogether, these features underscore the model’s strong potential as an animal platform for in-depth, physiologically relevant investigations of human microbiome–gut–organ axes.

## Figures and Tables

**Table 1:** An overview of major human donor, sample handling, recipient, and engraftment parameters in the Human microbiota-associated(HMA) pig model.

Classification	Characteristics	Specification	*n*	%
Donor	Cohort size	1 cohort	16	69.6
		2 cohort	6	26.1
		5 cohort	1	4.3
	Number per cohort	1	15	65.2
		3	1	4.4
		4	2	8.7
		5	3	13.0
		10	2	8.7
	Age group * (years)	Infant (0–1 y)	16	61.5
		Toddler (1–3 y)	1	3.8
		Children and adolesents (4–18 y)	3	11.5
		Adult (≥19 y)	6	23.2
	Healthy status	Healthy	18	78.3
		Specific phenotype	5	21.7
	Exclusion criteria specified	Yes	11	47.8
		No	12	52.2
Sample inocula	Materials used in processing	Fresh	0	0
		Frozen	23	100
	Vehicle solution in dilution	Phosphate-buffered saline (PBS)	5	21.7
		Pre-reduced PBS	7	30.4
		PBS with cystein	8	34.8
		Infant formula	2	8.7
		Not specified	1	4.4
	Sample purification	Centrifugation	4	17.4
		Filtration	1	4.3
		Not specified	18	78.3
	Storage Agent	Glycerol	22	95.7
		Not specified	1	4.3
	Sample pooling	Pooled	16	69.6
		Not pooled	7	30.4
Recipient pig and FMT regimen	Status	Germ-free	22	95.7
		AIMD	1	4.3
	Species	Purebred	5	21.7
		Crossbred	11	47.8
		Not specified	7	30.5
	Age at first inoculation	≤1 day after birth	6	26.1
		Postnatal days 2–8	12	52.2
		Postnatal days 9–14	2	8.7
		Postnatal week >2	3	13.0
	FMT route	Oral	23	100
	FMT dose	<1 mL	2	8.7
		1 mL	7	30.4
		2 mL	7	30.4
		>2 mL	5	21.7
		Not specified	2	8.7
	FMT duration	Single	13	56.5
		Multiple	10	43.5
Engraftment	Assessment **	Perfomed	9	39.1
		Not performed	14	60.9

* Age-group counts exceed the study counts because some studies include multiple donor age groups; ** Engraftment assessment refers to the qualitative and quantitative similarity of donor-derived taxa between human inoculum and recipient pigs, as determined by 16S rRNA gene sequencing, wherein the analysis is specifically limited to engraftment attributable to human FMT and not to other experimental manipulations. Quantitative analyses compared the taxa relative abundances based on sequencing read counts before and after FMT, whereas qualitative analyses indicate the presence or absence of taxa following transplantation; AIMD: Antibiotic-induced microbiota depletion, FMT: Fecal microbiota transplantation.

**Table 2:** Recommended reporting items on the HMA pig model.

Category	Items to Be Mentioned
Human donor	□ Basic information: Sex, age at fecal sampling, Body mass index (BMI) □ Cohort information: No. of an independent cohort, no. of human donors per cohort □ Physiological phenotype: Presence of obesity- or diabetes-associated metabolic traits (Yes/No) □ Dietary pattern at time of stool collection: Western, Mediterranean, East-Asian, Vegan, High fat high sucrose □ Donor lifestyle: Physically active, Moderately active, Sedentary □ Diagnosed disease and drug treatment history: Name of disease, Duration of diagnosis, type of medication, and treatment period □ Safety screening: Antibiotic resistance genes (ARGs) and zoonotic pathogens □ Inclusion and exclusion criteria: May include factors such as antibiotic use, chronic disease, gastrointestinal disturbances, and medication use
Recipient pig	□ Basic information: Pig strains, sex, age at FMT □ Recipient allocation: Number of recipient pigs per inoculum □ Pig preparation strategy: Germ-free; AIMD □ Construction of AIMD model: Age at exposure; antibiotic regimen; dose; route, duration, washout period □ Verification of AIMD: Pre- and post-treatment changes in microbial abundance, alpha, and beta diversity assessed by 16s rRNA amplicon sequencing □ Housing: Housing condition, no. of pigs in the cages, feed type and feeding method
Inoculum preparations	□ Materials used in processing: Fresh (Prepared immediately post-defecation without freezing)/Frozen (Prepared from frozen–thawed samples) □ Fecal sampling and transportation: Sample weight, storage method, duration, temperature □ Dilution: Suspension buffer, concentration □ Anaerobic handling: Use of anaerobic supplements (e.g., L-cysteine) or pre-reduced media □ Purification: Centrifugation; filtration; sedimentation □ Storage: Cryoprotectant, temperature, duration □ FMT Administration: Route, dose, duration □ Pooling strategy: Yes (Samples pooled across different individuals or across different time points from the same donor)/ No
Engraftment	□ Sample information: Pig age at observation, collection sites □ Index: Identity and number of colonizing taxa, together with their relative abundances at the OTU/ASV level in the human inoculum and recipient pigs □ Omics data: Transcriptomic, proteomic, and metabolomic datasets may be consulted

## Data Availability

No new data were generated in this study. Data used for reanalysis and visualization were derived from previously published studies and their Supplementary Materials.

## Supplementary Materials

The following supporting information can be downloaded at: https://www.mdpi.com/article/10.3390/ijms27041987/s1.

## Abbreviations

The following abbreviations are used in this manuscript:

FMT	Fecal microbiota transplantation
SPF	Specific pathogen-free
HMA	Human microbiota associated
PRISMA	Preferred Reporting Items for Systematic Reviews and Meta-Analyses
PRIM	Preferred Reporting Items
DOAJ	Directory of Open Access Journals
ARGs	Antibiotic resistance genes
PBS	Phosphate-buffered saline
OTUs	Operational taxonomic units
GF	Germ-free
AIMD	Antibiotic-induced microbiota depletion
UIFM	Urban infant fecal microbiota
RIFM	Rural infant fecal microbiota
HHGM	Healthy human gut microbiota
UHGM	Unhealthy human gut microbiota
EcN	*Escherichia coli* Nissle 1917
LGG	*Lactobacillus rhamnosus* GG
HBC	Hyperimmune bovine colostrum
PCM	Protein-calorie malnutrition
NK cells	Natural killer cells
DCs	Dendritic cells
Th cells	Helper T cells
ACE	Angiotensin-converting enzyme
IAV	Influenza A virus
oHFM	Obese human fecal microbiota
hHFM	Healthy human fecal microbiota
HRV	Rotavirus
SCFA	Short-chain fatty acid
