# Supplementary material for: Human Microbiota-Associated Pig Models for Translational Microbiome Research: A Scoping Review
Source: Int J Mol Sci. 2026 Feb 19;27(4):1987. doi: 10.3390/ijms27041987 (PMC12940510; doi:10.3390/ijms27041987)
Supplement: Supplementary file 1 [file ijms-27-01987-s001.zip › Supplementary material S1_0219.pdf]

Supplementary material S1

1. Preferred Reporting Items for Systematic reviews and Meta-Analyses extension for Scoping Reviews (PRISMA-ScR) Checklist

Section	Item	PRISMA-ScR Checklist Item	Reported Section
**Title**			
Title	1	Identify the report as a scoping review.	**Title**
**Abstract**			
Structured summary	2	Provide a structured summary that includes (as applicable) background, objectives, eligibility criteria, sources of evidence, charting methods, results, and conclusions that relates to the review questions and objectives	**2**
**Introduction**			
Rationale	3	Describe the rationale for the review in the context of what is already known. Explain why the review questions/objectives lend themselves to a scoping review approach.	**2**
Objectives	4	Provide an explicit statement of the questions and objectives being addressed with reference to their key elements (e.g., population or participants, concepts, and context) or other relevant key elements used to conceptualize the review questions and/or objectives.	**2**
**Methods**			
Protocol and registration	5	Indicate whether a review protocol exists; state if and where it can be accessed (e.g., a Web address); and if available, provide registration information, including the registration number.	**2**
Eligibility criteria	6	Specify characteristics of the sources of evidence used as eligibility criteria (e.g., years considered, language, and publication status), and provide a rationale.	**2**
Information sources*	7	Describe all information sources in the search (e.g., databases with dates of coverage and contact with authors to identify additional sources), as well as the date the most recent search was executed.	**2**
Search	8	Present the full electronic search strategy for at least 1 database, including any limits used, such that it could be repeated.	**2**
Selection of sources of evidence†	9	State the process for selecting sources of evidence (i.e., screening and eligibility) included in the scoping review.	**2**
Data charting process‡	10	Describe the methods of charting data from the included sources of evidence (e.g., calibrated forms or forms that have been tested by the team before their use, and whether data charting was done independently or in duplicate) and any processes for obtaining and confirming data from investigators.	**2**<br>**6.4**
Data items	11	List and define all variables for which data were sought and any assumptions and simplifications made.	**2**
Critical appraisal of individual sources of evidence§	12	If done, provide a rationale for conducting a critical appraisal of included sources of evidence; describe the methods used and how this information was used in any data synthesis (if appropriate).	**No**
Synthesis of results	13	Describe the methods of handling and summarizing the data that were charted	**2**<br>**4**
**Results**			
Selection of sources of evidence	14	Give numbers of sources of evidence screened, assessed for eligibility, and included in the review, with reasons for	**2**<br>**4**

		exclusions at each stage, ideally using a flow diagram.	
Characteristics of sources of evidence	15	For each source of evidence, present characteristics for which data were charted and provide the citations.	**3**
Critical appraisal within sources of evidence	16	If done, present data on critical appraisal of included sources of evidence (see item 12).	**No**
Results of individual sources of evidence	17	For each included source of evidence, present the relevant data that were charted that relate to the review questions and objectives.	**3.1-3.5**<br>**4.1-4.5**
Synthesis of results	18	Summarize and/or present the charting results as they relate to the review questions and objectives.	**3.1-3.5**<br>**4.1-4.5**
**Discussion**			
Summary of evidence	19	Summarize the main results (including an overview of concepts, themes, and types of evidence available), link to the review questions and objectives, and consider the relevance to key groups.	**3.5**<br>**4.5**
Limitations	20	Discuss the limitations of the scoping review process.	**6.4**
Conclusions	21	Provide a general interpretation of the results with respect to the review questions and objectives, as well as potential implications and/or next steps.	**7**
**Funding**			
Funding	22	Describe sources of funding for the included sources of evidence, as well as sources of funding for the scoping review. Describe the role of the funders of the scoping review.	**Funding**

JBI = Joanna Briggs Institute; PRISMA-ScR = Preferred Reporting Items for Systematic reviews and Meta-Analyses extension for Scoping Reviews.

* Where sources of evidence (see second footnote) are compiled from, such as bibliographic databases, social media platforms, and Web sites.

† A more inclusive/heterogeneous term used to account for the different types of evidence or data sources (e.g., quantitative and/or qualitative research, expert opinion, and policy documents) that may be eligible in a scoping review as opposed to only studies. This is not to be confused with information sources (see first footnote).

‡ The frameworks by Arksey and O'Malley (6) and Levac and colleagues (7) and the JBI guidance (4, 5) refer to the process of data extraction in a scoping review as data charting.

§ The process of systematically examining research evidence to assess its validity, results, and relevance before using it to inform a decision. This term is used for items 12 and 19 instead of "risk of bias" (which is more applicable to systematic reviews of interventions) to include and acknowledge the various sources of evidence that may be used in a scoping review (e.g., quantitative and/or qualitative research, expert opinion, and policy document).

From: Tricco AC, Lillie E, Zarin W, O'Brien KK, Colquhoun H, Levac D, et al. PRISMA Extension for Scoping Reviews (PRISMAScR): Checklist and Explanation. Ann Intern Med. 2018;169:467–473. doi: 10.7326/M18-0850.

2. Eligible criteria

Identification	Inclusion criteria	Exclusion criteria
**Publication Type**	• Original research articles	• Reviews, case series, case reports, clinical guidelines, conference abstracts, letters, preprints, note and editorials
**Research field**	• Foundational research describing the establishment of HMA pig models<br>• HMA pig Studies to investigate microbiome dynamics, host physiological responses, and underlying mechanisms.	• Studies using other animal models<br>• Studies not involving microbiome or microbiota analyses
**Screening**	**Inclusion criteria**	**Exclusion criteria**
**Donor**	• Studies involving any human donor type were eligible, with no restrictions on donor age, health status, or pooling strategy.	
**Sample**	• Human derived whole fecal microbiota	• Studies based on artificially reconstructed bacterial consortia or defined subsets of human-derived taxa, rather than whole fecal microbiota
**Recipient Pig**	• GF or antibiotic-depleted pigs colonized with human gut microbiota	• Studies involving SPF or conventional pigs
**F/W* duration**	• No minimum post-FMT follow-up duration was required, and studies were included regardless of the length of follow-up.	-
**Engraftment assessment**	• Studies enabling quantitative and qualitative comparison of taxa before and after FMT<br>• Using 16s rRNA amplicon profiling	• Studies lacking paired pre-/post-FMT taxonomic data<br>• Using DNA fingerprinting–based profiling

F/W: Follow up; GF: Germ-free; SPF: Specific pathogen free; FMT: Fecal microbiota transplantation;

3. Pubmed (2025.9.28, 2026.1.8)

Query (2007-2025)	Results
("Swine"[Mesh] OR pig* OR piglet* OR porcine OR swine) AND ("Germ-Free Life"[Mesh] OR germ-free OR germfree OR gnotobiotic* OR humanized OR "microbiota humanization" OR "human microbiota associated" OR HMA OR "human flora-associated" OR HFA OR "human gut microbiota" OR "human fecal microbiota" OR "antibiotic treated" OR "antibiotic-treated" OR "antibiotic depleted" OR "antibiotic-depleted" OR "antibiotic-induced depletion") AND (transplant* OR inoculat* OR coloniz* OR engraft* OR establish* OR "fecal transplant*" OR "fecal microbiota transplant*" OR FMT OR "stool transplant*" OR “assessment”)	521

4. Web of Science (2025.9.28, 2026.1.8)

Query (2007-2025)	Results
(pig* OR piglet* OR porcine OR swine) AND (germ-free OR gnotobiotic* OR "microbiota humanization" OR humanized OR "human microbiota associated" OR HMA OR "human flora-associated" OR HFA OR "human gut microbiota" OR "human fecal microbiota" OR "antibiotic treated" OR "antibiotic-treated" OR "antibiotic depleted" OR "antibiotic-depleted" OR "antibiotic-induced depletion") AND (transplant* OR inoculat* OR coloniz* OR engraft* OR establish* OR "fecal transplant*" OR "fecal microbiota transplant*" OR FMT OR "stool transplant*" OR "assessment")	604

5. Scopus (2025.9.28, 2026.1.8)

Query (2007-2025)	Results
(pig* OR piglet* OR porcine OR swine) AND ("germ free" OR germ-free OR gnotobiotic* OR "microbiota humanization" OR humanized OR "human microbiota associated" OR HMA OR "human flora associated" OR HFA OR "human gut microbiota" OR "human fecal microbiota" OR "antibiotic treated" OR "antibiotic-treated" OR "antibiotic depleted" OR "antibiotic-depleted" OR "antibiotic induced depletion") AND (transplant* OR inoculat* OR coloniz* OR engraft* OR establish* OR "fecal transplant*" OR "fecal microbiota transplant*" OR FMT OR "stool transplant*" OR assessment)	647

6. DOAJ search (2025.9.28, 2026.1.8)

Query (2007-2025)	Results
**"microbiota" AND "humanization"**	**12**
