# Supplementary material for: Human Microbiota-Associated Pig Models for Translational Microbiome Research: A Scoping Review
Source: Int J Mol Sci. 2026 Feb 19;27(4):1987. doi: 10.3390/ijms27041987 (PMC12940510; doi:10.3390/ijms27041987)
Supplement: Supplementary file 1 [file ijms-27-01987-s001.zip › Supplementary material S2_0219.pdf]

Supplementary material S2

1. Data processing and visualization for Figure 3

The figure was reconstructed using the Excel datasets provided in Supplementary Tables 7 and 10 of Aluthge et al. [11]. The original datasets reported the number, mean relative abundance, and standard deviation (SD) of core ASVs at the phylum, family, and genus levels across human donors, mice, and piglets. Because pigs were used as recipients only for donors #1, 2, 3, 4, and 8, we extracted the mean relative abundance and SD values for each phylum from these donors. For R-based analysis, phyla were organized in rows, with mean relative abundance and SD values provided separately for human donor samples and recipient piglet samples. The extracted data were then visualized using R to generate **Figure 3**.

2. R code for generating Figure 3

```
# Load required packages

library(tidyverse)

library(readxl)


# 1. Set file path

file_path <- file.choose()

print(paste("Selected file:", file_path))


# 2. Read and preprocess data from each sheet

read_and_process_sheet <- function(sheet_name) {

  data <- read_excel(file_path, sheet = sheet_name)


  donor_num <- str_extract(sheet_name, "\\d+")


  processed_data <- data %>%

    slice(-1) %>%   # Remove header row if present

    rename(

      Phylum = 1,

      Human_Mean = 2,

      Human_SD = 3,

      Piglet_Mean = 4,

      Piglet_SD = 5

    ) %>%
```

```r
    mutate(across(-Phylum, as.numeric)) %>%
    mutate(Donor = paste("Donor", donor_num))

  return(processed_data)
}

# 3. Read all donor sheets
sheet_names <- c("Donor_1", "Donor_2", "Donor_3", "Donor_4", "Donor_8")
all_data <- map_df(sheet_names, read_and_process_sheet)

# 4. Convert data to long format
data_long <- all_data %>%
  pivot_longer(
    cols = c(Human_Mean, Piglet_Mean),
    names_to = "Group",
    values_to = "Mean_Abundance"
  ) %>%
  mutate(
    SD = case_when(
      Group == "Human_Mean" ~ Human_SD,
      Group == "Piglet_Mean" ~ Piglet_SD
    ),
    Group = factor(
      Group,
      levels = c("Human_Mean", "Piglet_Mean"),
      labels = c("Human", "Piglet")
    ),
    Donor = factor(Donor, levels = paste("Donor", c(1, 2, 3, 4, 8)))
  )

# 5. Calculate descriptive statistics
descriptive_stats <- data_long %>%
  group_by(Phylum, Group) %>%
```

```
  summarise(
    n = n(),
    Mean = mean(Mean_Abundance),
    SD = sd(Mean_Abundance),
    .groups = "drop"
  )

# Inspect descriptive statistics
print(descriptive_stats)

# 6. Box plot with jittered points and sample size annotation
box_dot_with_stats <- ggplot(
  data_long,
  aes(x = Group, y = Mean_Abundance, fill = Group)
) +
  geom_boxplot(alpha = 0.6, outlier.shape = NA, width = 0.5) +
  geom_point(
    aes(color = Donor, shape = Donor),
    position = position_jitter(width = 0.15),
    size = 3,
    alpha = 0.8
  ) +
  # Annotate sample size for each group
  geom_text(
    data = descriptive_stats,
    aes(x = Group, y = -0.02, label = paste0("n=", n)),
    vjust = 1,
    size = 3,
    color = "gray40",
    inherit.aes = FALSE
  ) +
  facet_wrap(~ Phylum, ncol = 3, scales = "free_y") +
  scale_fill_manual(values = c("Human" = "#2E86AB", "Piglet" = "#A23B72")) +
```

```
  scale_color_brewer(palette = "Dark2") +
  scale_shape_manual(values = c(16, 17, 15, 18, 8)) +
  labs(
    title = "Distribution of Phylum-Level Mean Relative Abundance",
    x = "Group",
    y = "Mean Relative Abundance"
  ) +
  theme_bw() +
  theme(axis.text.x = element_text(angle = 45, hjust = 1))

print(box_dot_with_stats)
```

3. Data processing and visualization for Figure 4(A)

The figure was reconstructed using the Excel datasets provided in Supplementary Tables 7 and 10 of Aluthge et al. [11]. The original datasets reported the number, mean relative abundance, and standard deviation (SD) of core ASVs at the phylum, family, and genus levels across human donors, mice, and piglets. Because pigs were used as recipients only for donors #1, 2, 3, 4, and 8, we extracted the mean relative abundance and SD values for each family from these donors. For R-based analysis, families were organized in rows, with mean relative abundance and SD values provided separately for human donor samples and recipient piglet samples. The extracted data were then visualized using R to generate **Figure 4(A)**.

4. R code for generating Figure 4(A)

```
# ============================================================
# Bubble plot (Family) from an Excel file you select
# - Reads donor sheets (Donor_1 ~ Donor_8) in the same format:
#     Genus/Family | Humans | (SD cols) | Piglets | (SD cols)
# - Computes RAW fold change (Piglets / Humans)
# - Makes bubble plot for Top 15 families by mean Human abundance
# - Saves outputs (PNG/PDF)
# ============================================================

rm(list = ls())
gc()
```

```
# 1) Install & load packages --------------------------------
pkgs <- c("readxl", "dplyr", "tidyr", "stringr", "purrr", "ggplot2")
for (p in pkgs) {
  if (!requireNamespace(p, quietly = TRUE)) install.packages(p)
}
library(readxl)
library(dplyr)
library(tidyr)
library(stringr)
library(purrr)
library(ggplot2)

# 2) Choose Excel file ----------------------------------------
file_path <- file.choose()
message("Selected file: ", file_path)
out_dir <- dirname(file_path)

# 3) Identify donor sheets ------------------------------------
all_sheets <- excel_sheets(file_path)
donor_sheets <- all_sheets[grepl("^Donor_", all_sheets, ignore.case = TRUE)]
if (length(donor_sheets) == 0) stop("No donor sheets found (sheet names like 'Donor_1',
'Donor_2', ...).")
message("Donor sheets: ", paste(donor_sheets, collapse = ", "))

# 4) Helper: parse numeric safely ----------------------------
parse_num <- function(x) {
  x <- as.character(x)
  x <- str_replace_all(x, ",", "")
  x <- str_replace_all(x, "<", "") # e.g., "<0.0001" -> "0.0001"
  suppressWarnings(as.numeric(x))
}

# 5) Read + merge all donors (as text -> numeric) ------------
```

```
all_data <- map_dfr(donor_sheets, function(sh) {

  df <- read_excel(file_path, sheet = sh, col_types = "text")
  names(df) <- str_trim(names(df))

  # First column should be Family (or Genus). We'll treat it as Family here.
  if (!("Family" %in% names(df))) names(df)[1] <- "Family"

  # Find Humans / Piglets mean columns by header names
  humans_col  <- names(df)[tolower(names(df)) == "humans"]
  piglets_col <- names(df)[tolower(names(df)) == "piglets"]

  if (length(humans_col) != 1 || length(piglets_col) != 1) {
    warning("Sheet ", sh, ": Could not uniquely identify 'Humans' and 'Piglets' columns. Skipped.")
    return(NULL)
  }

  df %>%
    transmute(
      Family = as.character(.data$Family),
      Human_Abundance  = parse_num(.data[[humans_col]]),
      Piglet_Abundance = parse_num(.data[[piglets_col]]),
      Donor = sh
    ) %>%
    filter(!is.na(Family), Family != "", !Family %in% c("Family", "Genus")) %>%
    mutate(
      Human_Abundance  = ifelse(is.na(Human_Abundance), 0, Human_Abundance),
      Piglet_Abundance = ifelse(is.na(Piglet_Abundance), 0, Piglet_Abundance)
    )
})

message("Merged rows: ", nrow(all_data))
print(head(all_data, 10))
```

```
# 6) RAW fold change (Piglet/Human) --------------------------
# NOTE: This matches your "raw FC" logic, but avoids forcing 10 arbitrarily.
# - Human=0 & Piglet=0 -> NA (undefined)
# - Human=0 & Piglet>0 -> Inf
# - Else -> Piglet/Human
all_data <- all_data %>%
  mutate(
    Fold_Change = case_when(
      Human_Abundance == 0 & Piglet_Abundance == 0 ~ NA_real_,
      Human_Abundance == 0 & Piglet_Abundance   > 0 ~ Inf,
      TRUE ~ Piglet_Abundance / Human_Abundance
    )
  )

# 7) Change categories (with ranges in labels) ---------------
cat_levels <- c(
  "Strongly Decreased (<0.5x)",
  "Moderately Decreased (0.5-0.8x)",
  "Stable (0.8-1.2x)",
  "Moderately Increased (1.2-2x)",
  "Strongly Increased (>2x)"
)

all_data <- all_data %>%
  mutate(
    Change_Category = case_when(
      is.na(Fold_Change) ~ NA_character_,
      Fold_Change < 0.5 ~ "Strongly Decreased (<0.5x)",
      Fold_Change >= 0.5 & Fold_Change < 0.8 ~ "Moderately Decreased (0.5-0.8x)",
      Fold_Change >= 0.8 & Fold_Change <= 1.2 ~ "Stable (0.8-1.2x)",
      Fold_Change > 1.2 & Fold_Change <= 2 ~ "Moderately Increased (1.2-2x)",
      Fold_Change > 2 ~ "Strongly Increased (>2x)",
```

```
      TRUE ~ NA_character_
    ),
    Change_Category = factor(Change_Category, levels = cat_levels)
  )

# 8) Donor order -------------------------------------------
# If you want Donor_1..Donor_8 order even when some are missing:
donor_order <- paste0("Donor_", c(1, 2, 3, 4, 5, 6, 7, 8))
present_donors <- donor_order[donor_order %in% unique(all_data$Donor)]
all_data$Donor <- factor(all_data$Donor, levels = present_donors)

# 9) Select top 15 families by mean Human abundance -----------
top_families <- all_data %>%
  group_by(Family) %>%
  summarise(Mean_Human = mean(Human_Abundance, na.rm = TRUE), .groups = "drop") %>%
  arrange(desc(Mean_Human)) %>%
  slice_head(n = 15) %>%
  pull(Family)

filtered_data <- all_data %>% filter(Family %in% top_families)

# Order families by mean human abundance (desc)
family_order <- filtered_data %>%
  group_by(Family) %>%
  summarise(Mean_Human = mean(Human_Abundance, na.rm = TRUE), .groups = "drop") %>%
  arrange(desc(Mean_Human)) %>%
  pull(Family)

filtered_data$Family <- factor(filtered_data$Family, levels = family_order)

# 10) Color palette -----------------------------------------
category_colors <- c(
  "Strongly Decreased (<0.5x)" = "#1f78b4",
```

```
  "Moderately Decreased (0.5-0.8x)" = "#a6cee3",
  "Stable (0.8-1.2x)" = "#33a02c",
  "Moderately Increased (1.2-2x)" = "#fb9a99",
  "Strongly Increased (>2x)" = "#e31a1c"
)

# 11) Bubble plot (size = Human abundance %) -----------------
p_percent <- ggplot(filtered_data, aes(x = Donor, y = Family)) +
  geom_point(aes(size = Human_Abundance * 100, color = Change_Category),
             alpha = 0.85, stroke = 0.4) +
  scale_size_continuous(
    name = "Human Abundance (%)",
    range = c(2, 12),
    breaks = c(0.1, 1, 5, 20, 50),
    limits = c(0, 55),
    labels = c("0.1%", "1%", "5%", "20%", "50%")
  ) +
  scale_color_manual(
    name = "Change Category",
    values = category_colors,
    drop = FALSE
  ) +
  labs(
    title = "Major Family Abundance Changes: Human vs Piglet",
    subtitle = "Point size indicates human abundance (%); color indicates raw FC (Piglet/Human)",
    x = "Donor",
    y = "Family"
  ) +
  theme_minimal() +
  theme(
    plot.title = element_text(hjust = 0.5, face = "bold", size = 14),
    plot.subtitle = element_text(hjust = 0.5, size = 10, margin = margin(b = 12)),
    axis.text.x = element_text(angle = 45, hjust = 1, size = 9, face = "bold"),
```

```
    axis.text.y = element_text(size = 8, face = "bold"),
    axis.title = element_text(face = "bold", size = 11),
    legend.title = element_text(face = "bold", size = 9),
    legend.text = element_text(size = 8),
    legend.position = "right",
    panel.grid.major = element_line(color = "grey85"),
    panel.grid.minor = element_blank(),
    panel.border = element_rect(color = "grey70", fill = NA, linewidth = 0.5),
    plot.background = element_rect(fill = "white", color = NA),
    plot.margin = margin(1, 1, 1, 1, "cm")
  ) +
  guides(
    color = guide_legend(override.aes = list(size = 4), order = 1),
    size  = guide_legend(order = 2)
  )

print(p_percent)

# 12) Save plot --------------------------------------------
out_png <- file.path(out_dir, "Figure_Family_Abundance_Changes_rawFC.png")
out_pdf <- file.path(out_dir, "Figure_Family_Abundance_Changes_rawFC.pdf")

ggsave(out_png, p_percent, width = 12, height = 9, dpi = 300, bg = "white")
ggsave(out_pdf, p_percent, width = 12, height = 9, bg = "white")

message("Saved:")
message(" - ", out_png)
message(" - ", out_pdf)

# 13) Summary stats ----------------------------------------
cat("\nTop 15 families based on mean human abundance:\n")
top_family_stats <- all_data %>%
  group_by(Family) %>%
```

```
  summarise(
    Mean_Human = mean(Human_Abundance, na.rm = TRUE),
    Mean_Human_Percent = mean(Human_Abundance * 100, na.rm = TRUE),
    Max_Human_Percent = max(Human_Abundance * 100, na.rm = TRUE),
    .groups = "drop"
  ) %>%
  arrange(desc(Mean_Human)) %>%
  slice_head(n = 15)

print(top_family_stats, n = 15)

cat("\nDistribution of change categories (top 15 families only):\n")
change_summary <- filtered_data %>%
  count(Change_Category) %>%
  mutate(Percentage = round(n / nrow(filtered_data) * 100, 1))
print(change_summary)

cat("\nDistribution across human abundance percentage ranges (top 15 families only):\n")
percent_distribution <- filtered_data %>%
  mutate(Percent_Category = case_when(
    Human_Abundance * 100 < 0.1 ~ "Below 0.1%",
    Human_Abundance * 100 >= 0.1 & Human_Abundance * 100 < 1 ~ "0.1–1%",
    Human_Abundance * 100 >= 1 & Human_Abundance * 100 < 5 ~ "1–5%",
    Human_Abundance * 100 >= 5 & Human_Abundance * 100 < 20 ~ "5–20%",
    Human_Abundance * 100 >= 20 & Human_Abundance * 100 < 50 ~ "20–50%",
    Human_Abundance * 100 >= 50 ~ "≥50%"
  )) %>%
  count(Percent_Category) %>%
  mutate(Percentage = round(n / nrow(filtered_data) * 100, 1))

print(percent_distribution)
```

5. Data processing and visualization for Figure 4(B)

The figure was reconstructed using the Excel datasets provided in Supplementary Tables 7 and 10 of Aluthge et al. [11]. The original datasets reported the number, mean relative abundance, and standard deviation (SD) of core ASVs at the phylum, family, and genus levels across human donors, mice, and piglets. Because pigs were used as recipients only for donors #1, 2, 3, 4, and 8, we extracted the mean relative abundance and SD values for each genus from these donors. For R-based analysis, genera were organized in rows, with mean relative abundance and SD values provided separately for human donor samples and recipient piglet samples. The extracted data were then visualized using R to generate **Figure 4(B)**.

6. R code for generating Figure 4(B)

```
## ============================================================

## Raw FC (no pseudocount) + Top 43 genera

## Barplot + Heatmap with category ranges in legend labels

## ============================================================


rm(list = ls())

gc()


suppressPackageStartupMessages({

  library(readxl)

  library(dplyr)

  library(stringr)

  library(purrr)

  library(ggplot2)

})


## ----------------------------

## 0) Settings

## ----------------------------

file_path <- file.choose()

out_dir <- dirname(file_path)


top_n_genus <- 43    # include exactly 43 genera


# Category labels with ranges (what you requested)
```

```
cat_labels <- c(
  "Strongly Decreased (<0.5x)",
  "Moderately Decreased (0.5–0.8x)",
  "Stable (0.8–1.2x)",
  "Moderately Increased (1.2–2x)",
  "Strongly Increased (>2x)"
)

# Ordered factor for consistent legend ordering
cat_order <- cat_labels

# Colors (you can change if you want)
bar_colors <- c(
  "Strongly Decreased (<0.5x)"          = "#1f77b4",
  "Moderately Decreased (0.5–0.8x)"     = "#aec7e8",
  "Stable (0.8–1.2x)"                   = "#2ca02c",
  "Moderately Increased (1.2–2x)"       = "#ffbb78",
  "Strongly Increased (>2x)"            = "#d62728"
)

heat_colors <- c(
  "Strongly Increased (>2x)"            = "#D73027",
  "Moderately Increased (1.2–2x)"       = "#FC8D59",
  "Stable (0.8–1.2x)"                   = "#FFFFBF",
  "Moderately Decreased (0.5–0.8x)"     = "#91BFDB",
  "Strongly Decreased (<0.5x)"          = "#4575B4"
)

## ----------------------------
## 1) Identify donor sheets
## ----------------------------
all_sheets <- excel_sheets(file_path)
donor_sheets <- all_sheets[grepl("^Donor_", all_sheets)]
```

```
message("Selected file: ", file_path)
message("Donor sheets used: ", paste(donor_sheets, collapse = ", "))

if (length(donor_sheets) == 0) stop("No donor sheets found (pattern '^Donor_').")

## ----------------------------
## 2) Read and merge donor data (robust)
##       - read as text to preserve scientific notation
##       - convert only Humans/Piglets with as.numeric()
## ----------------------------
all_data <- map_dfr(donor_sheets, function(sheet){

  df <- read_excel(file_path, sheet = sheet, col_types = "text")
  names(df) <- str_trim(names(df))

  # If the first column is ...1 in some sheets, force it to Genus
  if (!("Genus" %in% names(df))) names(df)[1] <- "Genus"

  humans_col   <- names(df)[tolower(names(df)) == "humans"]
  piglets_col <- names(df)[tolower(names(df)) == "piglets"]

  if (length(humans_col) != 1 || length(piglets_col) != 1) {
    warning("Sheet ", sheet, ": Humans/Piglets columns not found uniquely. Skipped.")
    return(NULL)
  }

  df %>%
    transmute(
      Genus = as.character(.data$Genus),
      Human_Mean   = as.numeric(.data[[humans_col]]),
      Piglet_Mean = as.numeric(.data[[piglets_col]]),
      Donor = sheet
    ) %>%
```

```r
    filter(!is.na(Genus), Genus != "", Genus != "Genus") %>%
    mutate(
      Human_Mean   = ifelse(is.na(Human_Mean), 0, Human_Mean),
      Piglet_Mean = ifelse(is.na(Piglet_Mean), 0, Piglet_Mean)
    )
})

message("Total rows after merge: ", nrow(all_data))
print(head(all_data, 10))

## ----------------------------
## 3) Compute raw FC (no pseudocount)
## ----------------------------
all_data <- all_data %>%
  mutate(
    Raw_FC = case_when(
      Human_Mean == 0 & Piglet_Mean == 0 ~ NA_real_,
      Human_Mean == 0 & Piglet_Mean   > 0 ~ Inf,
      TRUE ~ Piglet_Mean / Human_Mean
    )
  )

## ----------------------------
## 4) Assign categories WITH range labels
## ----------------------------
all_data <- all_data %>%
  mutate(
    ChangeCategory = case_when(
      is.na(Raw_FC) ~ NA_character_,
      Raw_FC < 0.5 ~ "Strongly Decreased (<0.5x)",
      Raw_FC >= 0.5 & Raw_FC < 0.8 ~ "Moderately Decreased (0.5–0.8x)",
      Raw_FC >= 0.8 & Raw_FC <= 1.2 ~ "Stable (0.8–1.2x)",
      Raw_FC > 1.2 & Raw_FC <= 2.0 ~ "Moderately Increased (1.2–2x)",
```

```
      Raw_FC > 2.0 ~ "Strongly Increased (>2x)",
      TRUE ~ NA_character_
    ),
    ChangeCategory = factor(ChangeCategory, levels = cat_order)
  )

## ----------------------------
## 5) Select EXACTLY 43 genera
##       - Choose by mean Human abundance across donors (most abundant in humans)
##       - Ensures both plots use same 43 genera
## ----------------------------
genus_rank <- all_data %>%
  group_by(Genus) %>%
  summarise(
    Human_Mean_Avg = mean(Human_Mean, na.rm = TRUE),
    .groups = "drop"
  ) %>%
  arrange(desc(Human_Mean_Avg)) %>%
  slice_head(n = top_n_genus)

top_genus <- genus_rank$Genus

data_43 <- all_data %>%
  filter(Genus %in% top_genus) %>%
  mutate(
    Genus = factor(Genus, levels = rev(top_genus)),
    Donor = factor(Donor, levels = donor_sheets)
  )

## ----------------------------
## 6) Barplot (mean raw FC across donors) for 43 genera
##       - Mean computed using finite FC values only (excludes Inf/NA)
## ----------------------------
```

```
genus_summary_43 <- data_43 %>%
  group_by(Genus) %>%
  summarise(
    Mean_Raw_FC = mean(Raw_FC[is.finite(Raw_FC)], na.rm = TRUE),
    Any_Inf = any(is.infinite(Raw_FC)),
    .groups = "drop"
  ) %>%
  mutate(
    ChangeCategory = case_when(
      is.na(Mean_Raw_FC) ~ NA_character_,
      Mean_Raw_FC < 0.5 ~ "Strongly Decreased (<0.5x)",
      Mean_Raw_FC >= 0.5 & Mean_Raw_FC < 0.8 ~ "Moderately Decreased (0.5–0.8x)",
      Mean_Raw_FC >= 0.8 & Mean_Raw_FC <= 1.2 ~ "Stable (0.8–1.2x)",
      Mean_Raw_FC > 1.2 & Mean_Raw_FC <= 2.0 ~ "Moderately Increased (1.2–2x)",
      Mean_Raw_FC > 2.0 ~ "Strongly Increased (>2x)",
      TRUE ~ NA_character_
    ),
    ChangeCategory = factor(ChangeCategory, levels = cat_order)
  )

p_bar <- ggplot(
  genus_summary_43,
  aes(
    x = Mean_Raw_FC,
    y = Genus,
    fill = ChangeCategory
  )
) +
  geom_col() +
  scale_fill_manual(values = bar_colors, drop = FALSE) +
  geom_vline(xintercept = c(0.5, 0.8, 1.2, 2.0), linetype = "dashed", alpha = 0.5) +
  labs(
    title = paste0("Genus Abundance Changes (Top ", top_n_genus, " genera; Raw FC)"),
```

```
    subtitle = "Mean raw fold change across donors (Piglet/Human; Inf excluded from mean)",
    x = "Mean Raw Fold Change (Piglet / Human)",
    y = "Genus",
    fill = "Change Category"
  ) +
  theme_minimal() +
  theme(
    axis.text.y = element_text(size = 7),
    legend.position = "right",
    plot.title = element_text(hjust = 0.5, face = "bold"),
    plot.subtitle = element_text(hjust = 0.5)
  )

print(p_bar)

## -----------------------------
## 7) Heatmap (43 genera) with FC labels
## -----------------------------
heatmap_43 <- data_43 %>%
  mutate(
    Display_Label = case_when(
      is.na(Raw_FC) ~ "",
      is.infinite(Raw_FC) ~ "Inf",
      Raw_FC == 0 ~ "0.0",
      Raw_FC < 0.1 ~ "<0.1x",
      Raw_FC > 100 ~ ">100x",
      TRUE ~ sprintf("%.1fx", Raw_FC)
    )
  )

p_heat <- ggplot(heatmap_43, aes(x = Donor, y = Genus, fill = ChangeCategory)) +
  geom_tile(color = "white", linewidth = 0.5) +
  geom_text(aes(label = Display_Label), size = 2.5, color = "black", fontface = "bold") +
```

```
  scale_fill_manual(values = heat_colors, drop = FALSE) +
  labs(
    title = paste0("Genus Abundance Changes Heatmap (Top ", top_n_genus, " genera; Raw FC)"),
    subtitle = "Per donor; labels indicate raw fold change (Piglet/Human)",
    x = "Donor",
    y = "Genus",
    fill = "Change Category"
  ) +
  theme_minimal() +
  theme(
    axis.text.x = element_text(angle = 45, hjust = 1, face = "bold"),
    axis.text.y = element_text(size = 8),
    panel.grid = element_blank(),
    legend.position = "right",
    plot.title = element_text(hjust = 0.5, face = "bold"),
    plot.subtitle = element_text(hjust = 0.5)
  )

print(p_heat)

## -----------------------------
## 8) Save outputs (PDF + PNG)
## -----------------------------
bar_pdf   <- file.path(out_dir, paste0("Genus_Top", top_n_genus, "_RawFC_Barplot.pdf"))
bar_png   <- file.path(out_dir, paste0("Genus_Top", top_n_genus, "_RawFC_Barplot.png"))
heat_pdf <- file.path(out_dir, paste0("Genus_Top", top_n_genus, "_RawFC_Heatmap.pdf"))
heat_png <- file.path(out_dir, paste0("Genus_Top", top_n_genus, "_RawFC_Heatmap.png"))

ggsave(bar_pdf,   p_bar,   width = 9, height = 11, units = "in")
ggsave(bar_png,   p_bar,   width = 9, height = 11, units = "in", dpi = 300)
ggsave(heat_pdf, p_heat, width = 7, height = 9,   units = "in")
ggsave(heat_png, p_heat, width = 7, height = 9,   units = "in", dpi = 300)
```

```
message("Saved files:")
message(" - ", bar_pdf)
message(" - ", bar_png)
message(" - ", heat_pdf)
message(" - ", heat_png)

## -----------------------------
## (Optional) Save tables
## -----------------------------
# write.csv(data_43, file.path(out_dir, paste0("all_data_top", top_n_genus, "_rawFC.csv")), row.names = FALSE)
# write.csv(genus_summary_43, file.path(out_dir, paste0("genus_summary_top", top_n_genus, "_rawFC.csv")), row.names = FALSE)
```

7. Data processing and visualization for Figure 5

Two supplementary datasets from Dhakal et al. [38] (Table S2b and Table S3b) were used in this reanalysis. These datasets report OTU occurrence and taxonomic assignments for urban and rural infant fecal microbiota (UIFM and RIFM) and the corresponding transplanted piglets across five gastrointestinal sites (ileum mucosa, ileum digesta, colon mucosa, colon digesta, and feces at necropsy). Occurrence values were retained, while parentheses indicating the number of detected samples were removed, and the data were reorganized in Excel to generate new datasets. In this reanalysis, an OTU was considered present if it was detected in at least one sample within a given donor group or intestinal region (the original study included five human donors and four recipient piglets). This structure enabled systematic identification of shared, donor-specific, and recipient-specific OTUs and their relative contributions. The processed datasets were saved as separate Excel sheets (RIFM_OTU and UIFM_OTU) and subsequently analyzed in R using the code provided below.

8. R code for generating Figure 5

```
library(readxl)
library(dplyr)
library(tidyr)


# ========================
# 0. Select input file
# ========================
file_path <- file.choose()


# ========================
# 1. Common function for occurrence comparison
# ========================
compare_occurrence_raw <- function(df, donor_col, compare_cols) {

  results <- list()

  for (comp in compare_cols) {

    # ---- Shared OTUs between donor and comparison region ----
    both_present <- df %>%
      filter(
        !is.na(.data[[donor_col]]), .data[[donor_col]] > 0,
```

```
    !is.na(.data[[comp]]), .data[[comp]] > 0
  )

both_present_count <- nrow(both_present)

both_present_donor_sum <- sum(both_present[[donor_col]], na.rm = TRUE)
both_present_compare_sum <- sum(both_present[[comp]], na.rm = TRUE)

# ---- OTUs uniquely detected in the comparison region ----
new_in_compare <- df %>%
  filter(
    (is.na(.data[[donor_col]]) | .data[[donor_col]] == 0),
    !is.na(.data[[comp]]), .data[[comp]] > 0
  )

new_in_compare_count <- nrow(new_in_compare)

total_compare_sum <- df %>%
  filter(!is.na(.data[[comp]]), .data[[comp]] > 0) %>%
  summarise(s = sum(.data[[comp]], na.rm = TRUE)) %>%
  pull(s)

new_in_compare_sum <- sum(new_in_compare[[comp]], na.rm = TRUE)

new_in_compare_pct <- round(new_in_compare_sum / total_compare_sum * 100, 2)

# ---- Store results ----
results[[comp]] <- data.frame(
  Comparison_Column = comp,
  Both_Present_Count = both_present_count,
  Both_Present_Donor_Sum = round(both_present_donor_sum, 2),
  Both_Present_Compare_Sum = round(both_present_compare_sum, 2),
  New_In_Compare_Count = new_in_compare_count,
```

```
      New_In_Compare_Sum = new_in_compare_pct
    )
  }

  bind_rows(results)
}

# =========================
# RIFM analysis
# =========================
rifm <- read_excel(file_path, sheet = "RIFM_OTU")

rifm_compare_cols <- c(
  "Ileum mucosa",
  "Ileum digesta",
  "Colon mucosa",
  "Colon digesta",
  "Pig Feces"
)

rifm_result <- compare_occurrence_raw(
  df = rifm,
  donor_col = "RIFM",
  compare_cols = rifm_compare_cols
)

print(rifm_result)

# =========================
# UIFM analysis
# =========================
uifm <- read_excel(file_path, sheet = "UIFM_OTU")
```

```
uifm_compare_cols <- c(
  "Ileum mucosa",
  "Ileum digesta",
  "Colon mucosa",
  "Colon digesta",
  "Feces at necropsy"
)

uifm_result <- compare_occurrence_raw(
  df = uifm,
  donor_col = "UIFM",
  compare_cols = uifm_compare_cols
)

print(uifm_result)
```

9. Tables Generated from the R Code Analysis

Shared and RIFMP-specific OTUs: counts and relative abundance

Comparison_ Column	Both_Present_ Count	Both_Present _RIFM_Sum	Both_Present _Compare_Sum	New_In_Compare_ Count	New_In_Compare_ Sum
Ileum mucosa	97	37.79	79.644	71	18.164
Ileum digesta	64	29.515	76.332	61	21.925
Colon mucosa	101	36.997	82.589	51	11.56
Colon digesta	106	39.918	81.971	45	9.794
Feces at necropsy	102	40.809	82.064	66	11.44

Shared and UIFMP-specific OTUs: counts and relative abundance

Comparison_ Column	Both_Present_ Count	Both_Present _UIFM_Sum	Both_Present _Compare_Sum	New_In_Compare_ Count	New_In_Compare_ Sum
Ileum mucosa	90	71.819	76.197	51	20.585
Ileum digesta	64	56.634	85.515	21	11.888
Colon mucosa	81	68.098	52.132	33	39.975
Colon digesta	77	63.112	57.388	23	32.635
Feces at necropsy	78	62.882	59.851	31	31.597
