# Supplementary material for: Human Microbiota-Associated Pig Models for Translational Microbiome Research: A Scoping Review
Source: Int J Mol Sci. 2026 Feb 19;27(4):1987. doi: 10.3390/ijms27041987 (PMC12940510; doi:10.3390/ijms27041987)
Supplement: Supplementary file 1 [file ijms-27-01987-s001.zip › Supplementary table_0219.pdf]

Supplementary table S1. Human donor characteristics in HMA pig studies

References	Donor cohort*	No. of donor/cohort**	Age groups***	Health status of donors****	Human donor Exclusion criteria	Detailed exclusion criteria mentioned
Pang et al., 2007 [27]	1	1	Children and adolescent	Healthy	Specified	• No diarrhea or other digestive disorders<br>• No medication for at least 6 months
Che et al., 2008 [22]	1	1	Adult	Healthy	Specified	• No recent history of gastrointestinal disturbances<br>• No using antimicrobial agent
Shen et al., 2010 [28]	1	1	Adult	Healthy	Specified	• No signs of digestive disorders<br>• No medication for at least 6 months
Zhang et al., 2013 [29]	2	1 or 10	Infant<br>Adult	Healthy	Not specified	
Sponseller et al. 2015 [30]	1	10	Adult	Healthy	Not specified	
Wen et al., 2014 [31]	1	1	Infant	Healthy	Specified	• No signs of digestive disorders<br>• No medication prior to stool sample collection.
Zhang et al., 2014 [32]	1	1	Infant	Healthy	Not specified	
Twitchell et al., 2016 [33]	2	1	Infant	Specific phenotype	Specified	• No contraindication to receive Rotavirus vaccine<br>• No chronic health conditions, blood transfusion
Wang et al., 2016 [34]	1	1	Infant	Healthy	Specified	• No signs of digestive disorders<br>• No medication prior to stool sample collection.
Fischer et al., 2017 [104]	1	1	Infant	Healthy	Not specified	
Vlasova et al., 2017 [35]	1	1	Infant	Healthy	Not specified	
Kumar et al. 2018 [36]	1	1	Infant	Healthy	Specified	• Neither infant nor mother had any recent history of disease or antibiotic treatment
Miyazaki et al., 2018 [37]	1	1	Infant	Healthy	Not specified	
Dhakal et al., 2019 [38]	2	5	Infant	Specific phenotype	Not specified	

Lei et al., 2019 [39]	1	1	Infant	Healthy	Specified	• No contraindication to receive Rotavirus vaccine<br>• No chronic health conditions, blood transfusion<br>• Pathogen-free
Aluthge et al., 2020 [11]	5	1	Infant, Children and adolescent, Adult	Healthy	Specified	• Free of antibiotic use during the previous 6 mon.<br>• Non-antibiotic medication for a respiratory illness.
Michael et al., 2020 [40]	1	1	Infant	Healthy	Not specified	
Michael et al., 2021 [41]	1	1	Infant	Healthy	Not specified	
Renu et al. 2022 [42]	2	5	Children and adolescent	Specific phenotype	Not specified	
Michael et al. 2022 [43]	1	1	Infant	Healthy	Not specified	
Schrock et al. 2024 [44]	2	5	Infant	Specific phenotype	Not specified	
Zhang et al., 2025 [45]	1	4	Adult	Healthy	Specified	• No history of chronic diseases;<br>• No antiviral, antibacterial and fungal drugs in the past 3 months;<br>• Not eaten pork outside the large animal base in the past 2 weeks<br>• No constipation, diarrhea or blood in the stool within a week;
Amimo et al., 2025 [46]	2	3	Toddler	Specific phenotype	Specified	• No gastrointestinal infections and acute gastrointestinal infection

The ***Donor cohort** and ****No. of donors per cohort** columns refer, with respect to the recipient pigs, to the number of distinct donor cohorts administered and the number of donors included in each cohort, respectively; *****Age groups** are defined as follows: infants, 0–1 year; toddlers, 1–3 years; children and adolescents, 4–18 years; and adults, ≥19 years; The ******Health status of donors** column indicates whether each study used healthy donors or donors with specific phenotypes.

Supplementary table S2. Sample handling and preparation characteristics in HMA pig studies

References	Materials used in processing*	Sample collection	Storage agent	Storage condition	Vesicle solution In dilution	Diluted concentration	Purification method	Sample pooling**
Pang et al., 2007 [27]	Frozen	Not specified	10% glycerol	-70℃	PBS	1:20	Not specified	Not pooled
Che et al., 2008 [22]	Frozen	Not specified	10% glycerol	-80℃	PBS	1:20	Not specified	Not pooled
Shen et al., 2010 [28]	Frozen	Not specified	10% glycerol	-80℃	Pre-reduced PBS	1:20	Not specified	Not pooled
Zhang et al., 2013 [29]	Frozen	Airtight container	10% glycerol	-80℃	Pre-reduced PBS	1:10	Filtration	pooled
Sponseller et al. 2015 [30]	Frozen	Anaerobic chamber	10% glycerol	-80℃	Pre-reduced PBS	1:10	Not specified	pooled
Wen et al., 2014 [31]	Frozen	Not specified	15% glycerol	-80℃	Pre-reduced PBS	1:20	Centrifuged	Pooled
Zhang et al., 2014 [32]	Frozen	Not specified	15% glycerol	-80℃	Pre-reduced PBS	1:20	Not specified	Pooled
Twitchell et al., 2016 [33]	Frozen	Not specified	15% glycerol	-80℃	Pre-reduced PBS	1:20	Centrifuged	Not pooled
Wang et al., 2016 [34]	Frozen	Not specified	15% glycerol	-80℃	Pre-reduced PBS	1:20	Centrifuged	pooled
Fischer et al., 2017 [104]	Frozen	Not specified	30% glycerol	-80℃	PBS with cystein	1:20	Not specified	Pooled
Vlasova et al., 2017 [35]	Frozen	Not specified	30% glycerol	-80℃	PBS with cystein	1:20	Not specified	Pooled
Kumar et al. 2018 [36]	Frozen	Sterile cup	30% glycerol	-80℃	PBS with cystein	1:20	Not specified	Pooled
Miyazaki et al., 2018 [37]	Frozen	Not specified	30% glycerol	-80℃	PBS with cystein	1:20	Not specified	Pooled
Dhakal et al., 2019 [38]	Frozen	Sterile bottle	15% glycerol	-80℃	Infant formula	Not specified	Not specified	pooled
Lei et al., 2019 [39]	Frozen	Not specified	15% glycerol	-80℃	PBS	1:20	Centrifuged	Not pooled

Aluthge et al., 2020 [11]	Frozen	Not specified	50% glycerol	-80℃	Infant formula	1:20	Not specified	pooled
Michael et al., 2020 [40]	Frozen	Not specified	30% glycerol	-80℃	PBS with cystein	1:20	Not specified	Pooled
Michael et al., 2021 [41]	Frozen	Not specified	30% glycerol	-80℃	PBS with cystein	1:20	Not specified	Pooled
Renu et al. 2022 [42]	Frozen	Sterile bottle	15% glycerol	-80℃	PBS	Not specified	Not specified	pooled
Michael et al. 2022 [43]	Frozen	Not specified	30% glycerol	-80℃	PBS with cystein	1:20	Not specified	Not pooled
Schrock et al. 2024 [44]	Frozen	A tube	15% glycerol	-80℃	PBS	Not specified	Not specified	pooled
Zhang et al., 2025 [45]	Frozen	Sterile tube	Not specified	-80℃	Not mentioned	Not specified	Not specified	pooled
Amimo et al., 2025 [46]	Frozen	Sterile vial	30% glycerol	-80℃	PBS with cystein	1:50	Not specified	Not pooled

In the ***Materials used in processing** column, 'Fresh' indicates inocula prepared immediately after defecation without freezing, whereas 'Frozen' indicates inocula prepared from samples that were frozen and subsequently thawed; In the HMA pig studies reviewed, only **frozen samples** were used.; ****Pooling sample** refers to the combination of samples either from different individuals or from the same individual collected at multiple time points.; **PBS**, phosphate buffered saline

Supplementary table S3. Recipient pigs, FMT protocol characteristics in HMA pig studies

References	Status	Species	Feeding Regimen*	Number of recipient pigs per inoculum**	Age at FMT	Route of FMT	FMT dose	FMT duration
Pang et al., 2007 [27]	GF	Meishan	Sterilized milk and Infant cereal	28	12h after birth	Oral	1ml	10 days (Initial 3 consecutive days, then alternate days)
Che et al., 2008 [22]	GF	Meishan	Sterilized milk and Infant cereal	7	Newborn	Oral	1ml	7 consecutive days
Shen et al., 2010 [28]	GF	Meishan	Sterilized milk and Infant cereal	5	12 hours after birth	Oral	1ml	10 days (Initial 3 consecutive days, then alternate days)
Zhang et al., 2013 [29]	GF	Landrace and Yorkshire crossbred	Infant milk	2, 4	5d/8d/23d/30d after birth	Oral	3ml	Single day
Sponseller et al. 2015 [30]	GF	Not specified	Infant milk	4	5 days old	Oral	3ml	Single day
Wen et al., 2014 [31]	GF	Yorkshire crossbred	Not specified	4, 10, 12, 13	12 hours after birth	Oral	1ml	3 consecutive days
Zhang et al., 2014 [32]	GF	Landrace and Yorkshire crossbred	Sterilized milk	3, 4	12 hours after birth	Oral	1ml	3 consecutive days
Twitchell et al., 2016 [33]	GF	Yorkshire crossbred	Sterilized milk	5, 6, 7	5-7 days old	Oral	400-700 $\mu\ell$	Single day
Wang et al., 2016 [34]	GF	Yorkshire crossbred	Not specified	4	12 hours after birth	Oral	1ml	3 consecutive days
Fischer et al., 2017 [104]	GF	Landrace Yorkshire Duroc crossbred	Sterilized milk	3, 10, 18	4 days old	Oral	Not specified	Single day
Vlasova et al., 2017 [35]	GF	Landrace Yorkshire Duroc crossbred	Sterilized milk	2, 3, 5, 10	4 days old	Oral	2ml	Single day
Kumar et al. 2018 [36]	GF	Landrace Yorkshire Duroc crossbred	Sterilized milk	3, 5	4 days old	Oral	2ml	Single day

Miyazaki et al., 2018 [37]	GF	Yorkshire crossbred	Sterilized milk	11, 12	4 days old	Oral	2ml	Single day
Dhakal et al., 2019 [38]	GF	Not specified	Sterilized milk	4	2 weeks	Oral	5ml	Once weekly for 3 consecutive weeks
Lei et al., 2019 [39]	GF	Yorkshire	Sterilized milk	7, 11	4-5 days old	Oral	450 $\mu\ell$	2 consecutive days
Aluthge et al., 2020 [11]	GF	Landrace Duroc crossbred	Sterilized milk, Teklad 2919	3, 4	6w	Oral	4ml	2 doses, 2-week interval
Michael et al., 2020 [40]	GF	Not specified	Sterilized milk	11, 12	4 days old	Oral	2ml	Single day
Michael et al., 2021 [41]	GF	Not specified	Not specified	3, 4	4 days old	Oral	2ml	Single day
Renu et al. 2022 [42]	GF	Not specified	Infant milk	4-6	2 weeks	Oral	1ml	Single day
Michael et al. 2022 [43]	GF	Landrace Yorkshire Duroc crossbred	Not specified	6, 7	4 days old	Oral	2ml	Single day
Schrock et al. 2024 [44]	GF	Not specified	Sterilized milk	4-5	8 days old	Oral	5ml	Single day
Zhang et al., 2025 [45]	AIMD	Bama	Not specified	6	7–17 weeks, 18–27 weeks	Oral	Not specified	21 weeks (weekly)
Amimo et al., 2025 [46]	GF	Not specified	Sterilized milk	8, 11	6 days old	Oral	2ml	Single day

*In the **feeding regimen** column, sterilized milk refers to commercially available products, such as Similac (Abbott), Anyou (Huai'an), Hershey, and Parmalat. When sterility was not explicitly specified, the milk was categorized as infant milk.; ****Number of recipient pigs per inoculum** indicates the number of pigs in each group that received the same human-derived inoculum.; **GF**, germ-free; **AIMD**, antibiotic-induced microbiota depletion.

Supplementary table S4. Engraftment assessment parameters in HMA pig studies

References	Engraftment Assessment*	Assessement Method**	Brief description of engraftment efficiency***	Pig age at observation	Collection site	Profiling	Region	Taxonomic Level
Pang et al., 2007 [27]	Not performed							
Che et al., 2008 [22]	Not performed							
Shen et al., 2010 [28]	Not performed							
Zhang et al., 2013 [29]	Performed	Quantitative	Not specified	~ 48days	Colon, feces	16S	V6	Phyla
Sponseller et al. 2015 [30]	Performed	Quantitative	Not specified	~11days	feces	16S	V1-V2	Phyla
Wen et al., 2014 [31]	Not performed							
Zhang et al., 2014 [32]	Not performed							
Twitchell et al., 2016 [33]	Performed	Quantitative	Mean relative abundances were similar between UHGM pig samples and infant stool, whereas HHGM pig samples differed from their infant donors.	35days	Large intestinal contents	16S	V1-V2	Phyla
Wang et al., 2016 [34]	Not performed							
Fischer et al., 2017 [104]	Not performed							
Vlasova et al., 2017 [35]	Performed	Qualitative	The relative abundance of shared OTUs reached 99.94%	14days	feces	16S	V4-V5	Phyla, genus
Kumar et al. 2018 [36]	Performed	Qualitative Quantitative	Between 99.27% and 100% of cumulative OTUs in HIFM pig intestinal and fecal samples were present in the original HIFM.	11days	Intestinal tissues	16S	V4-V5	Phyla, genus
Miyazaki et al., 2018 [37]	Not performed							

Dhakal et al., 2019 [38]	Performed	Qualitative Quantitative	All phyla present in the inoculum were detected in fecal swabs from both piglet groups, with varying relative abundances.	~49days	Ileum, colon, feces	16S	V4	Phyla, OTUs
Lei et al., 2019 [39]	Not performed							
Aluthge et al., 2020 [11]	Performed	Qualitative Quantitative	Among the 27 core ASVs shared by three adult donors, 70–92% were detected at least once in piglets with only 22–74% of these ASVs consistently maintained (≥ 4 time points)	~71days	feces	16S	V4	Phyla, family ASV
Michael et al., 2020 [40]	Not performed							
Michael et al., 2021 [41]	Not performed							
Renu et al. 2022 [42]	Performed	Quantitative	The original HFM and the corresponding pig outgrowth HFM showed high compositional overlap (99% and 95%, respectively)	~49days	feces	16S	V4-V5	Genus
Michael et al. 2022 [43]	Not performed							
Schrock et al. 2024 [44]	Not performed							
Zhang et al., 2025 [45]	Performed	Qualitative	*Bacteroidia* and *Bacilli* in humanized pigs approximated human levels	~21weeks	feces	16S	V3-V4	Class
Amimo et al., 2025 [46]	Not performed							

* **Engraftment assessment** refers to the qualitative and quantitative similarity of donor-derived taxa between the human inoculum and recipient pigs, as determined by 16S rRNA gene sequencing, with the analysis specifically limited to engraftment attributable to human FMT and not to other experimental manipulations.; **Assessment method**: Quantitative analyses denote comparisons of taxa relative abundances derived from sequencing read counts across pre- and post-FMT samples, whereas qualitative analyses indicate the presence or absence of taxa following transplantation.; *****The Brief description of engraftment efficiency** column provides a concise summary of engraftment-related information as described in the original studies.
